# Physiological and genetic characterization of calcium phosphate precipitation by *Pseudomonas* species

**DOI:** 10.1038/s41598-018-28525-4

**Published:** 2018-07-05

**Authors:** Maxwell R. Fishman, Krista Giglio, David Fay, Melanie J. Filiatrault

**Affiliations:** 1 School of Integrated Plant Science, Section of Plant Pathology and Plant-Microbe Biology, Cornell University, Ithaca, NY 14853 United States; 2 Emerging Pests & Pathogens Research Unit, Robert W. Holley Center, USDA-Agricultural Research Service, Ithaca, NY 14853 United States

## Abstract

Microbial biomineralization is a widespread phenomenon. The ability to induce calcium precipitation around bacterial cells has been reported in several *Pseudomonas* species but has not been thoroughly tested. We assayed 14 *Pseudomonas* strains representing five different species for the ability to precipitate calcium. Calcium phosphate precipitated adjacent to the colonies of all the *Pseudomonas* strains tested and also precipitated on the surface of colonies for several of the *Pseudomonas* strains assayed. The precipitate was commonly precipitated as amorphous calcium phosphate, however seven of the 14 *Pseudomonas* strains tested precipitated amorphous apatite in agar adjacent to the colonies. Out of the seven *Pseudomonas* strains that precipitated amorphous apatite, six are plant pathogenic. The formation of amorphous apatite was commonly observed in the area of the agar where amorphous calcium phosphate had previously formed. A transposon mutagenesis screen in *Pseudomonas syringae* pv. tomato DC3000 revealed genes involved in general metabolism, lipopolysaccharide and cell wall biogenesis, and in regulation of virulence play a role in calcium precipitation. These results shed light on the common ability of *Pseudomonas* species to perform calcium precipitation and the underlying genetic regulation involved in biomineralization.

## Introduction

Biomineralization is the precipitation of inorganic minerals by biological organisms. In particular, bacteria readily perform biomineralization and the precipitation of calcium based minerals by bacteria are thought to be among the most common observed in nature^1^. Apatite is a calcium phosphate based mineral that is precipitated by a large number of bacteria including *Escherichia coli*, *Corynebacterium matruchotii*, *Ramlibacter tatouinensis*, *Streptococcus mutans*, *Pseudomonas aeruginosa*, *Pseudomonas fluorescens*, *Serattia marcescens*, *Streptococcus mutans* and *Streptococcus sanguis*^2–6^. Microbial apatite biomineralization occurs both extracellularly and intracellularly and is classified as either an active or passive process. The active process is called microbially controlled calcium precipitation (MCCP) and involves specific proteins or peptides and relies on a microbially produced matrix^7^. The passive process is thought to be a byproduct of bacterial metabolism and is referred to as microbially induced calcium precipitation (MICP)^8^. MICP is an extracellular process commonly initiated by a high concentration of calcium at a nucleation point on the cell surface^1^. Bacterial membranes and exopolysaccharides are major nucleation points for MICP^3,9,10^. An alkaline microenvironment occurs during MICP as a result of metabolic behavior of bacteria^1^. The end result of MICP is particles of different sizes with no set morphology^8^. In contrast to MICP, MCCP produces crystals of a common size and shape and is most commonly an intracellular process^7^. There are few examples of MCCP in bacteria, one being in the case of intracellular MCCP by *R. tataouinensis*^5^.

*Pseudomonas* species live in diverse environments and many are associated with higher organisms. The species *Pseudomonas putida*, *Pseudomonas fluorescens*, *Pseudomonas syringae*, *Pseudomonas savastanoi*, and *Pseudomonas viridiflava* commonly associate with plants. *P. putida* and *P. fluorescens* are rhizosphere-associated microbes that are considered plant-beneficial microorganisms^11,12^. In contrast, *P. syringae*, *P. savastanoi*, and *P. viridiflava* are foliar plant pathogens^13–15^. *Pseudomonas* is one of the many bacterial genera that is able to precipitate calcium. This has been observed as the precipitation of calcium carbonate in several ureolytic *Pseudomonas* species and in certain *P. aeruginosa* strains. In phosphate-sequestering *Pseudomonas* species, certain *P. aeruginosa* strains, and *P. fluorescens* strains, the precipitation of calcium results in apatite formation^6,16–20^. Calcium is abundant in and around plants. Inorganic calcium phosphate is found in the rhizosphere of plants and up to millimolar concentrations of calcium are found in the plant apoplast^21,22^. Calcium concentration increases in the apoplast of bean leaves during *P. syringae* pv. phaseolicola 1448a infection and in the xylem of tobacco plants during *Xyllela fastidiosa* infection^23,24^. It is currently unknown whether plant pathogenic *Pseudomonas* species can precipitate calcium when grown in high calcium environments.

In the current study, we demonstrate calcium precipitation by several plant-beneficial, rhizosphere-associated *Pseudomonas* species and by several plant-pathogenic, epiphytic *Pseudomonas* species. We show that this phenomenon occurs near neutral pH and that the spatial patterning and morphology of the calcium precipitate differs across species and strains. Lastly, we identify several genes that are involved in apatite biomineralization in *P*. *syringae* pv. tomato DC3000.

## Results

### *Pseudomonas* species increase the pH of medium during growth

**Figure 1:**
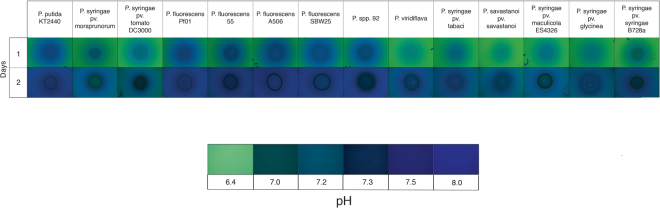
Pictures of *Pseudomonas* species grown on NB agar supplemented with calcium and 0.1% (w/v) BB over the course of two days. Pictures are of the same colony on day one and day two. The relative pH of the media can be determined by the change in the color of BB. Pictures of the color change in NB agar supplemented with calcium and 0.1% (w/v) BB from a pH of 6.4 to 8.0 is shown below the pictures of the bacterial colonies. This assay was repeated three separate times and the pictures are representative of those assays.

An increase in pH is commonly observed and thought to be necessary during MICP. Some *Pseudomonas* are reported to increase the pH of their immediate environment^25^. To determine if the 14 *Pseudomonas* species could increase the pH of the surrounding environment, bromothymol blue (BB) was used to monitor the change in pH during growth on nutrient broth (NB) agar plates. BB can detect changes in pH from a pH of 6.0 to a pH of 8.0. The initial pH of NB medium was 6.4 after calcium was added so any increase in pH should be detectable using BB. *Pseudomonas* colonies were monitored for two days after spotting on NB supplemented with Ca^2+^ and 0.1% (w/v) BB. All the *Pseudomonas* species assayed raised the pH of the surrounding medium to varying degrees, as determined by the blue color found adjacent to all of the bacterial colonies (Fig. 1). Some differences were observed between species as *P. syringae* pathovars, *P. viridiflava* NYS-1, and *P. savastanoi* pv. savastanoi 4352 produced a less alkaline environment than *P. putida* KT2440 and all the *P. fluorescens* strains assayed. In addition, the colonies of *P. syringae* pv. morsprunorum 5795, *P. syringae* pv. tomato DC3000, *P*. spp. 92, *P. viridiflava* NYS-1, *P. savastanoi* pv. savastanoi 4352, *P. syringae* pv. maculicola ES4326, and *P. syringae* pv. syringae B728a turned yellowish, suggesting the colony is a more acidic environment than the adjacent environment. This yellowish color was not observed in the other *Pseudomonas* strains assayed. Overall, since all the *Pseudomonas* species assayed increased the pH of the surrounding environment, they may possess the characteristics needed to precipitate calcium in the surrounding environment as well.

### Calcium precipitation among several *Pseudomonas* species

**Figure 2:**
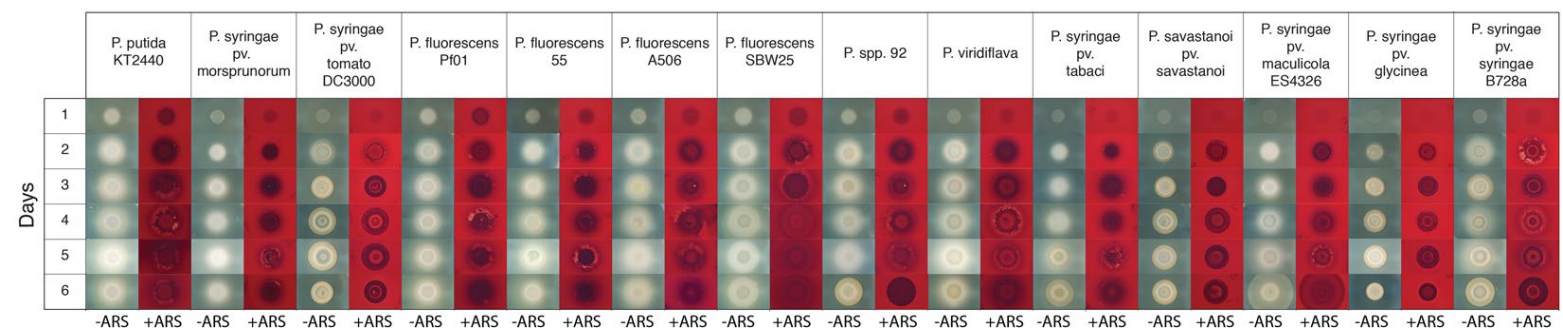
Pictures of *Pseudomonas* species grown on NB agar supplemented with calcium over the course of six days. Pictures were taken of the same colony before (−) and after (+) staining of the colonies with 1.0% (w/v) ARS. These pictures are representative of growth for each *Pseudomonas* species on NB agar with calcium. The assay was repeated five separate times and the pictures are representative of those assays.

The dye Alizarin Red S (ARS) was employed to quickly screen bacterial colonies for calcium precipitates. Within one day of growth on NB agar plates supplemented with Ca^2+^, ARS stained the agar around *Pseudomonas* colonies more heavily than other regions of the agar. This staining was visualized as a dark red area surrounding the colonies (Fig. 2). Prior to staining with ARS, these darkened areas were seen as a white halo in the agar around the bacterial colonies (Fig. 2). The staining of these white halos by ARS suggested they represented calcium rich areas. White halos did not form around bacterial colonies grown on NB agar plates without additional Ca^2+^ and bacterial colonies and agar on NB agar plates without additional Ca^2+^ added were not stained by ARS (Fig. S1). A white halo that could be stained by ARS persisted around *P. putida* KT2440 and *P. fluorescens* strains throughout six days, however the ARS-stained areas were occasionally dislodged during rinsing of the plates (Fig. 2). In contrast to *P. putida* KT2440 and the *P. fluorescens* strains, most of the *P. syringae* strains (except *P. syringae* pv. morsprunorum 5795) and *P*. spp. 92, had a calcium rich white halo form around the colonies that subsequently changed into a brown halo (Fig. 2). This transition from a white to brown halo occurred in *P. syringae* pv. tomato DC3000 after two days of growth (Fig. 2). ARS stained the brown halos, suggesting that they represent calcium rich areas as well.

ARS staining on the surface of colonies was prominent on *P. syringae* pv. morsprunorum 5795, *P. fluorescens* Pf0-1, *P. fluorescens* 55, *P. syringae* pv. tabaci ATCC11528, *P. savastanoi* pv. savastanoi 4352, and *P. syringae* pv. maculicola ES4326, suggesting that calcium is enriched on the surface of these *Pseudomonas* strains when grown on NB supplemented with Ca^2+^ (Fig. 2). ARS staining did not occur when strains were grown on NB agar plates lacking supplemental Ca^2+^ (Fig. S1). The colonies for *P. syringae* pv. tomato DC3000, *P. syringae* pv. glycinea 2159 Race 1, *P. savastanoi* pv. savastanoi 4325, and *P. syringae* pv. syringae B728a stained with ARS only after the brown halo was present (Fig. 2).

### Characterization of calcium phosphate precipitated by *Pseudomona*s species

**Figure 3:**
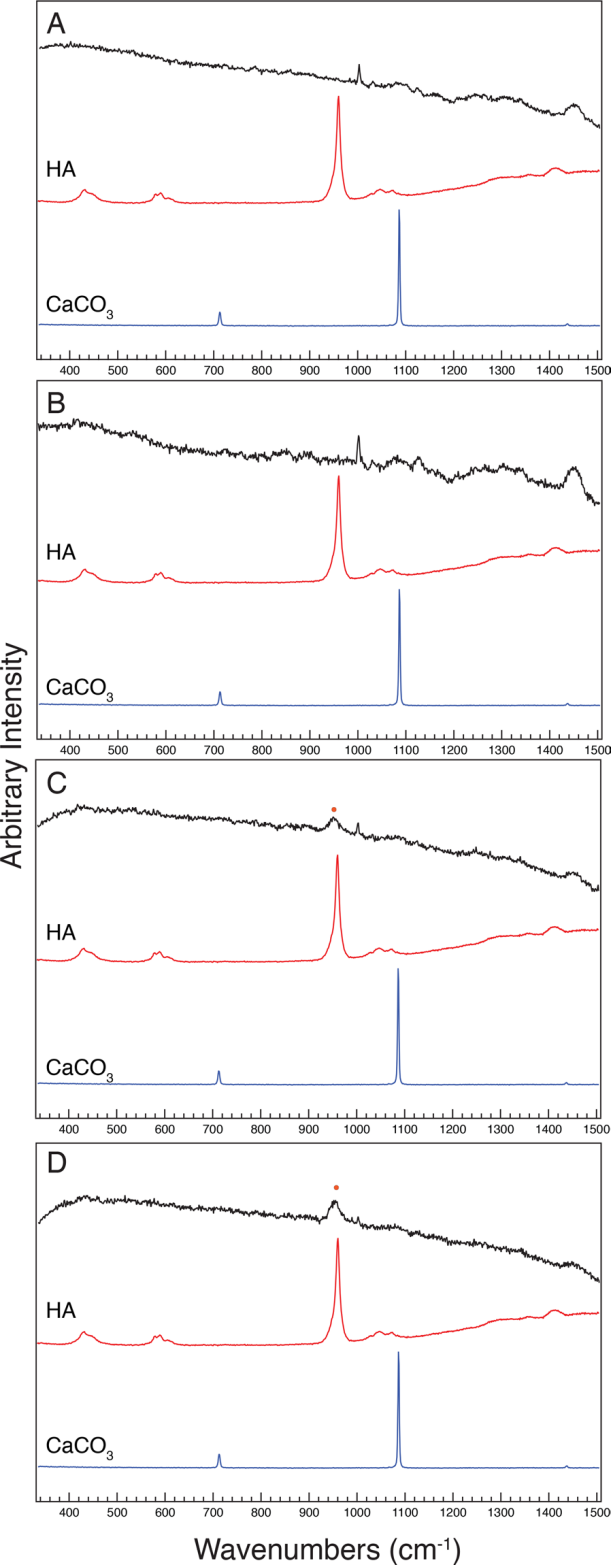
Raman spectra taken from the center of the surface of colonies of (**A**) *P. putida* KT2440, (**B**) *P. syringae* pv. morsprunorum 5795, (**C**) *P. syringae* pv. tomato DC3000, and (**D**) *P. fluorescens* Pf0-1, at six days of growth are colored black in each panel. A control for hydroxyapatite is labelled “HA” and is colored red and a control for calcium carbonate is labelled “CaCO_3_” and is colored blue. A broad peak slightly downshifted from 960 cm^−1^ is labelled with an orange dot and signifies the presence of amorphous calcium phosphate on the surface of cells. Bands commonly associated with biological organisms, including a peak for DNA (782 cm^−1^) and phenylalanine (1004 cm^−1^) and broad bands associated with amides (1230–1300 cm^−1^) and methyl groups (1430–1460 cm^−1^) can be seen on these spectra as well. This assay was performed three separate times and the spectra are representative of the assays. The y-axis is in arbitrary intensity units and the x-axis is in wavenumbers (cm^−1^).

**Figure 4:**
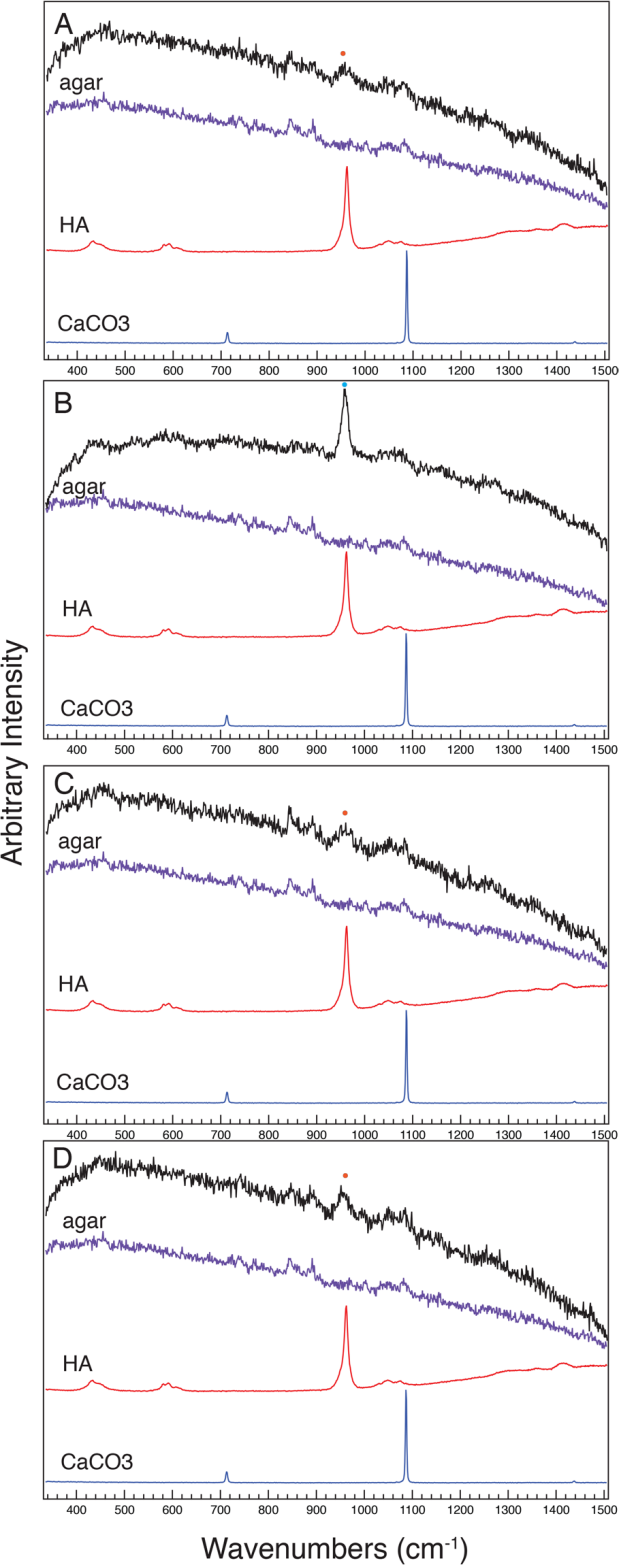
Raman spectra taken from the agar directly adjacent to live colonies of (**A**) *P. putida* KT2440, (**B**) *P. syringae* pv. morsprunorum 5795, (**C**) *P. syringae* pv. tomato DC3000, and (**D**) *P. fluorescens* Pf0-1, at six days of growth are colored black in each panel. A control spectrum for NB agar is labelled “agar” and colored in purple, a control spectrum for hydroxyapatite is labelled “HA” and colored in red, and a control spectrum for calcium carbonate is labelled “CaCO_3_” and is colored in blue. A broad peak centered around 955 cm^−1^ is labelled with an orange dot in the spectra for *P. putida* KT2440, *P. syringae* pv. morsprunorum 5795, and *P. fluorescens* Pf0-1 and reflects the presence of amorphous calcium phosphate in these samples. A sharper peak centered at 959 cm^−1^ in *P*. *syringae* pv. tomato DC3000 is labelled with a light blue dot and reflects the presence of amorphous apatite. The assay was repeated three independent times and the spectra are representative of those assays. The y-axis is in arbitrary intensity units and the x-axis is in wavenumbers (cm^−1^).

Since ARS could not distinguish if calcium was precipitated or in another form, such as chelated to a substrate, another method was required to further characterize the calcium rich areas. Spectroscopic techniques are able to distinguish whether a calcium precipitate, such as calcium phosphate or calcium carbonate, is present. Raman spectroscopy was employed as a noninvasive and nondestructive method that allowed for the same bacterial colony to be analyzed over the course of several days. After spotting cultures on agar plates supplemented with Ca^2+^, spectra were obtained from the center of the colonies and from the agar directly adjacent to the colonies (Figs 3, 4, Figs S2–S6). Spectra taken from the colonies of *P. syringae* pv. morsprunorum 5795, *P. fluorescens* Pf0-1, *P. fluorescens* 55, *P. fluorescens* A506, *P. syringae* pv. tabaci ATCC11528, and *P. syringae* pv. maculicola ES4326 exhibited a broad peak centered at 955 cm^−1^ within six days of growth (Figs 3, S2). Relative to the other peaks in the spectra, this peak increased over time for most of the *Pseudomonas* strains where it was detected (Fig. S3). Raman spectra taken from the surface of *P. savastanoi* pv. savastanoi 4352 colonies produced a relatively sharp peak centered at 959 cm^−1^ (Fig. S2). The sharpness and upshift of this peak suggests that the calcium precipitate on the cell surface of *P. savastanoi* pv. savastanoi 4352 is a different composition than the calcium precipitate on the cell surface of the other *Pseudomonas* strains. The broad peaks centered at 955 cm^−1^ and the sharp peak at 959 cm^−1^ are slightly downshifted from the expected peak at 961 cm^−1^ for hydroxyapatite (Figs 3, S3)^26^. A broad peak centered around 950-960 cm^−1^ is a standard peak associated with calcium phosphate and precipitation of this mineral on the cell surface is likely the cause of this peak in spectra from *P. syringae* pv. morsprunorum 5795, *P. fluorescens* Pf0-1, *P. fluorescens* 55, *P. fluorescens* A506, *P. syringae* pv. tabaci ATCC11528, and *P. syringae* pv. maculicola ES4326 colonies^27^. Since the peak from spectra produced by *P. savastanoi* pv. savastanoi 4352 was slightly lower than the expected peak for hydroxyapatite, the calcium precipitate on the cell surface of *P. savastanoi* pv. savastanoi 4352 likely represents amorphous apatite^26,27^. Overall, the Raman spectras indicate calcium phosphate precipitate occurred on the cell surface of six of the fourteen strains assayed. Surprisingly, no peaks for calcium carbonate were detected on the surface of any colonies (Figs 3, S2).

Exopolysaccharides (EPSs) produced by bacteria are considered nucleation points during calcium precipitation. *Pseudomonas* species produce the EPSs alginate and cellulose, which can act as nucleation points for calcium precipitation^28,29^. Raman spectra of alginate display several expected peaks below 1400 cm^−1^ and a broad peak centered at 3400 cm^−1^, while Raman spectra of cellulose display a sharp peak at 2900 cm^−1^^30–32^. In order to determine whether alginate and cellulose were present on bacterial colonies, Raman spectra from 335 cm^−1^ to 1515 cm^−1^ and from 2715 cm^−1^ to 3450 cm^−1^ were analyzed for peaks characteristic of alginate or cellulose from bacterial colonies grown on NB supplemented with Ca^2+^. (Figs 3, S3, S4). Within these spectra, none of the peaks associated with alginate or cellulose were detected. In the Raman spectra from 2715 cm^−1^ to 3450 cm^−1^, the only detected peaks were two broad peaks centered around 2872 cm^−1^ and 2946 cm^−1^ that correspond to the presence of lipids^33^. From this we conclude that alginate and cellulose are likely not abundant on the surface of any *Pseudomonas* colonies assayed.

As previously mentioned, on NB plates supplemented with Ca^2+^, the agar immediately adjacent to some colonies had a white or brown halo that stained with ARS. The spectra obtained from the agar where the white halos were present contained a small, broad peak that was centered at 955 cm^−1^ that likely represents amorphous calcium phosphate (Figs 4, S5, S6). In comparison, the spectra from the agar where the brown halos were present contained a strong peak centered at 959 cm^−1^ and likely represents amorphous apatite (Figs 4, S5, S6). These peaks were not present in the NB agar supplemented with Ca^2+^ control plate (Figs 4, S5, S6). Peaks associated with biological organisms were absent in the spectra taken from the agar adjacent to the cells, providing evidence that cells were not present in the surrounding halos. It should be noted that, *P. syringae* pv. maculicola ES4326 produced a brown halo in the agar on the sixth day of growth. However, the Raman spectra of the agar after six days of growth only had a small, broad peak with a center slightly upshifted from 955 cm^−1^ rather than a peak centered at 959 cm^−1^ as was seen with the other brown halos. The brown halo produced by *P. syringae* pv. maculicola ES4326 was more translucent than the brown halos produced by other strains. The translucent nature of the brown halo may have made it difficult for the Raman confocal microscope to detect a signal for amorphous apatite. Peaks expected for calcium carbonate were not observed in any of the samples (Figs 4, S5). Overall from these data, we conclude that all of the *Pseudomonas* strains assayed precipitate calcium phosphate in the adjacent environment, but the characteristics of the calcium phosphate differs between strains.

### Structure of calcium precipitates on the surface of *Pseudomonas* colonies

*P. putida* KT2440, *P. syringae* pv. morsprunorum 5795, *P. syringae* pv. tomato DC3000, and *P. fluorescens* Pf0-1 were analyzed using scanning electron microscopy (SEM) to determine the structure(s) of the calcium phosphate precipitated by these strains. These strains were chosen to further characterize via SEM because they represented three different *Pseudomonas* species and encompassed the different spatial patterns of calcium precipitation observed in the 14 *Pseudomonas* species tested.

**Figure 5:**
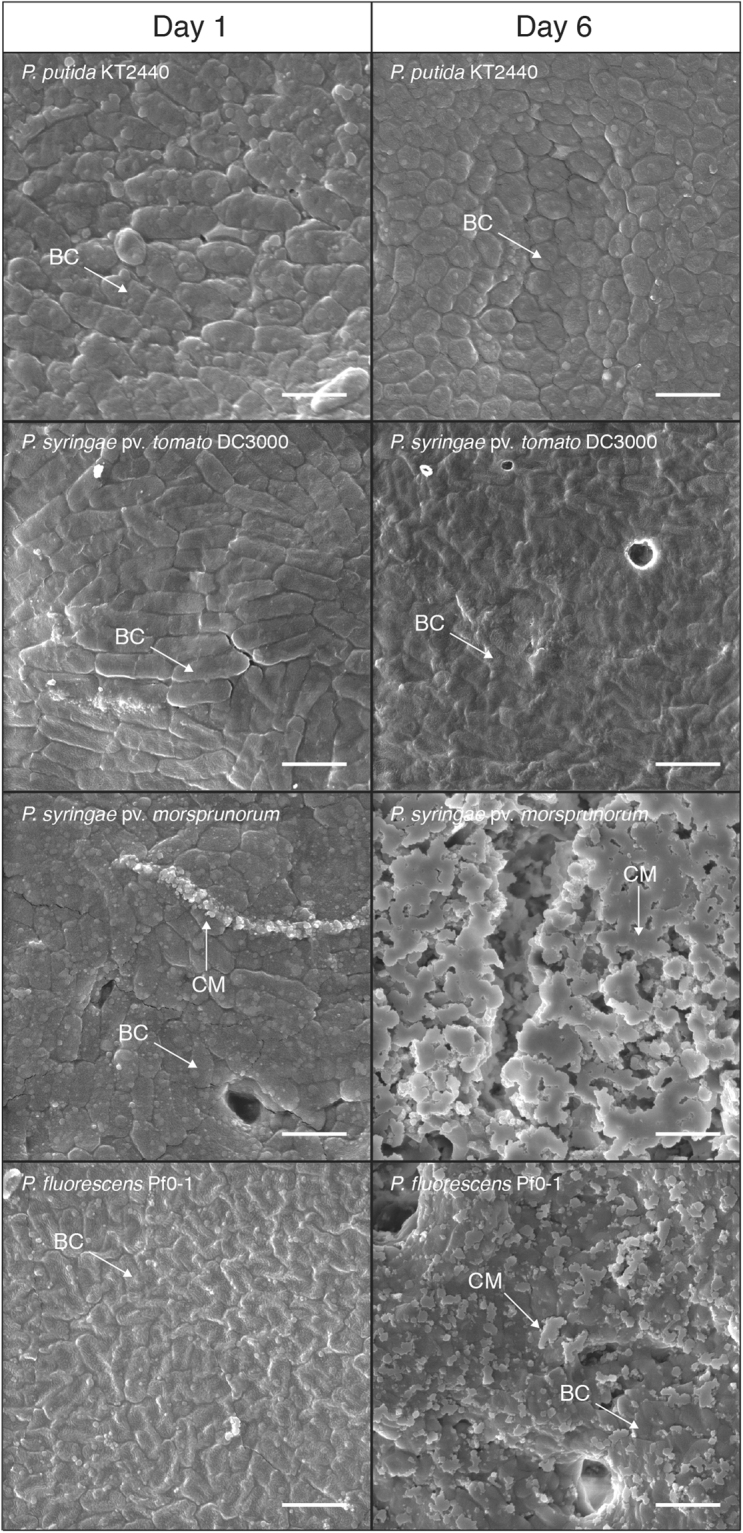
SEM images at 18,000x magnification of colon*y* surface for *P. putida* KT2440, *P. syringae* pv. morsprunorum 5795, *P. syringae* pv. tomato DC3000, and *P. fluorescens* Pf0-1 at 12 hours and six days of growth. 2 µm scale bars are indicated in the lower right-hand corner of the images. An arrow labelled “CM” points to calcium phosphate on the surface of cells are included on each image where calcium phosphate is thought to be present. An arrow labelled “BC” points to bacterial cells in each image. Imaging of cells with SEM was performed three times using separate biological replicates for each image. This image is representative of what was observed with the other replicates.

The strains were first observed after 12 hours of growth. By this time, several blebs appeared on the cell surface on all bacterial strains as well as small calcium phosphate particles on the surface of *P. syringae* pv. morsprunorum 5795 (Fig. 5). Subsequent observations of colonies after six days of growth showed that *P. putida* KT2440 had what appeared to be particles on the cell surface, *P. syringae* pv. tomato DC3000 did not appear to have any particles on the surface of cells, and *P. syringae* pv. morsprunorum 5795 and *P. fluorescens* Pf0-1 cell surfaces were partially mineralized (Fig. 5). The SEM data was consistent with the Raman spectra of the colonies.

### Structure of calcium precipitates in the agar adjacent to *Pseudomonas* colonies

**Figure 6:**
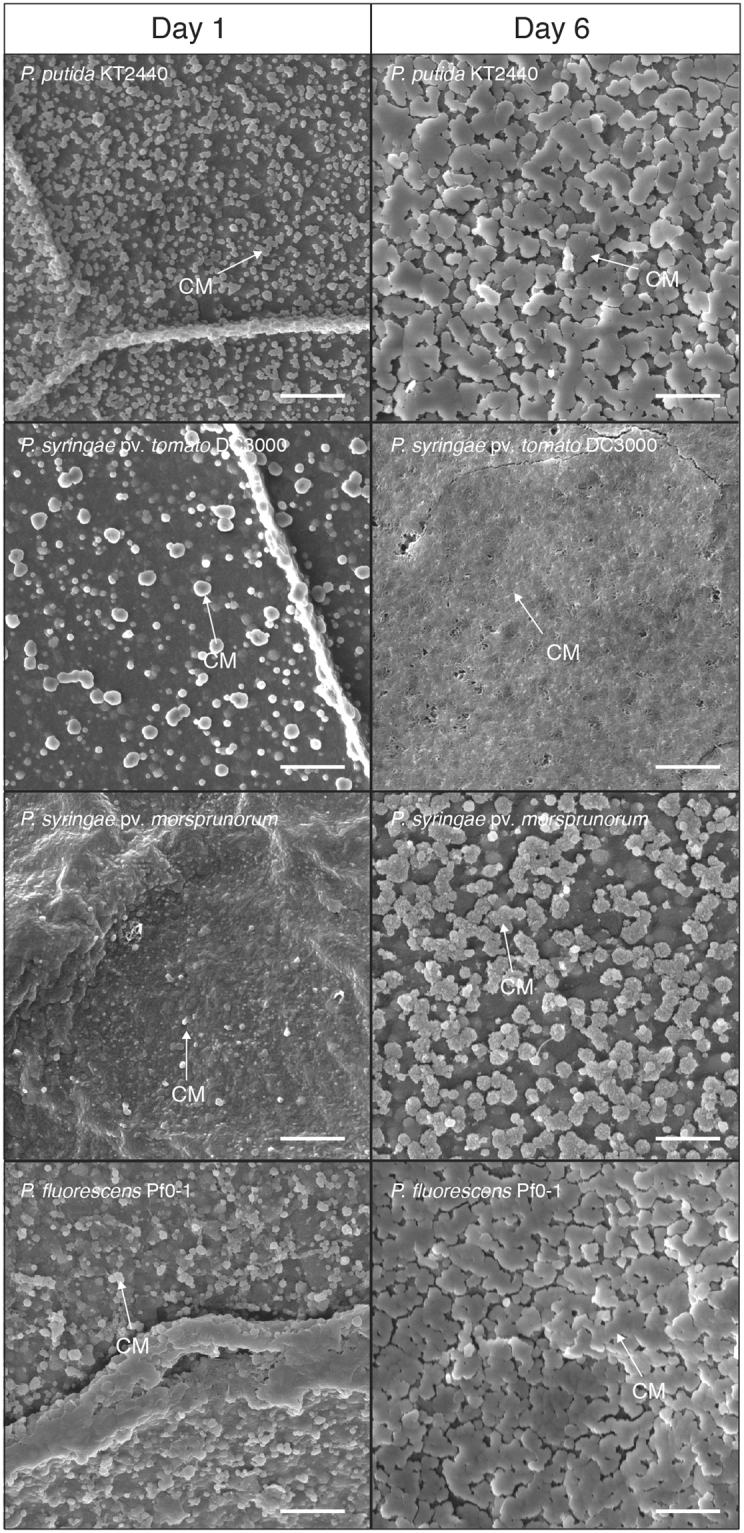
SEM images at 18,000x magnification of the agar adjacent to bacterial colonies for *P. putida* KT2440, *P. syringae* pv. morsprunorum 5795, *P. syringae* pv. tomato DC3000, and *P. fluorescens* Pf0-1 after 12 hours and six days of growth. 2 µm scale bars are indicated in the lower right-hand corner of the images. Arrows labelled “CM” points to calcium phosphate that has formed on the agar. Imaging of the agar adjacent to colonies was performed three times using three separate biological replicates. This image is representative of what was observed with the other replicates.

SEM analysis of the agar adjacent to the bacterial colonies showed that calcium phosphate particles formed next to *P. putida* KT2440, *P. fluorescens* Pf0-1, *P. syringae* pv. tomato DC3000, and *P. syringae* pv. morsprunorum 5795 colonies after 12 hours of growth (Fig. 6). These particles clustered around the bacterial colony and varied in size, shape, and number depending on the *Pseudomonas* strain. Sizes of the particles ranged from 200–400 nm long in the agar adjacent to *P. putida* KT2440 and *P. fluorescens* Pf0-1 cells, 200–600 nm long in the agar adjacent to *P. syringae* pv. tomato DC3000 cells, and 100–400 nm long in the agar adjacent to *P. syringae* pv. morsprunorum 5795 cells. Particles were present in the NB agar supplemented with Ca^2+^ control plate, however these particles were distinct from those that accumulated around bacterial colonies. Particles in the control plate were less numerous, smaller (100–200 nm in length), and diffuse throughout the entire agar plate instead of being heavily concentrated at a particular site (Fig. S7).

After six days of growth, larger calcium phosphate particles were seen via SEM in the agar adjacent to *P. putida* KT2440, *P. fluorescens* Pf0-1, and *P. syringae* pv. morsprunorum 5795 colonies (Fig. 6). The calcium phosphate precipitates on the agar adjacent to *P. putida* KT2440 and *P. fluorescens* Pf0-1 were around 2 µm long while calcium phosphate particles surrounding *P. syringae* pv. morsprunorum 5795 were numerous and around 600 nm long. The calcium precipitate that formed in the agar around *P. syringae* pv. tomato DC3000 was quite different in nature. By six days of growth, *P. syringae* pv. tomato DC3000 formed a brown halo of amorphous apatite around the colony (Fig. 2). This precipitate looked fibrous and porous at high magnification and the morphology was distinctly different from the precipitate formed by *P. putida* KT2440, *P. fluorescens* Pf0-1, or *P. syringae* pv. morsprunorum 5795 (Figs 6, S8). Overall from these data, we conclude that the calcium precipitates that formed in white and brown halos on the agar adjacent to bacterial colonies are not structured and have considerably different morphologies when compared to each other. This suggests that the precipitates are likely the product of MICP. However, the differing morphologies suggests that they are likely produced through different mechanisms.

### Identification of genes important for calcium precipitation of *P. syringae* pv. tomato DC3000

To better understand the genes involved in calcium precipitation in *Pseudomonas* species, we performed a transposon (Tn) mutagenesis screen using *P. syringae* pv. tomato DC3000 (*Pto*). *Pto* was chosen since it has a well annotated genome and produced a brown halo of amorphous apatite around the colonies. Our Tn mutagenesis screen resulted in approximately 55,000 Tn mutants. Of these, we selected 31 colonies that displayed an altered calcium precipitation phenotype. The phenotypes for each of the Tn mutants were re-tested for altered calcium precipitation as described in the methods section. The altered phenotypes included colonies that had: 1) increased amorphous apatite precipitation in the form of a brown halo that was darker or wider than WT, 2) reduced amorphous apatite precipitation that had a lighter brown halo as compared to WT or did not form a brown halo, or 3) altered calcium precipitation on the colony surface with or without a brown halo present in the agar (Fig. S9). Of the 31 strains that had altered calcium precipitation phenotypes, 14 were identical siblings after sequence determination of the Tn5 insertion site. One clone from each set of identical siblings was considered for further analysis. The site of the Tn insertion and the phenotype of the resultant set of 17 mutant strains that displayed altered calcium precipitation are summarized in Table S1. Growth curves were performed on the 17 mutant strains in NB medium and NB medium supplemented with Ca^2+^ (Fig. S10). It should be noted, several of the strains that had altered calcium precipitation or little to no visible amorphous apatite precipitation did not grow as well in NB whether or not there was Ca^2+^ supplementation. We categorized the 17 genes according to function and found that many genes encode for proteins related to general metabolism, cell wall and lipopolysaccharide homeostasis and transport, and pathogenesis (Table S1).

### RetS and TvrR regulate calcium phosphate precipitation in *Pto*

**Figure 7:**
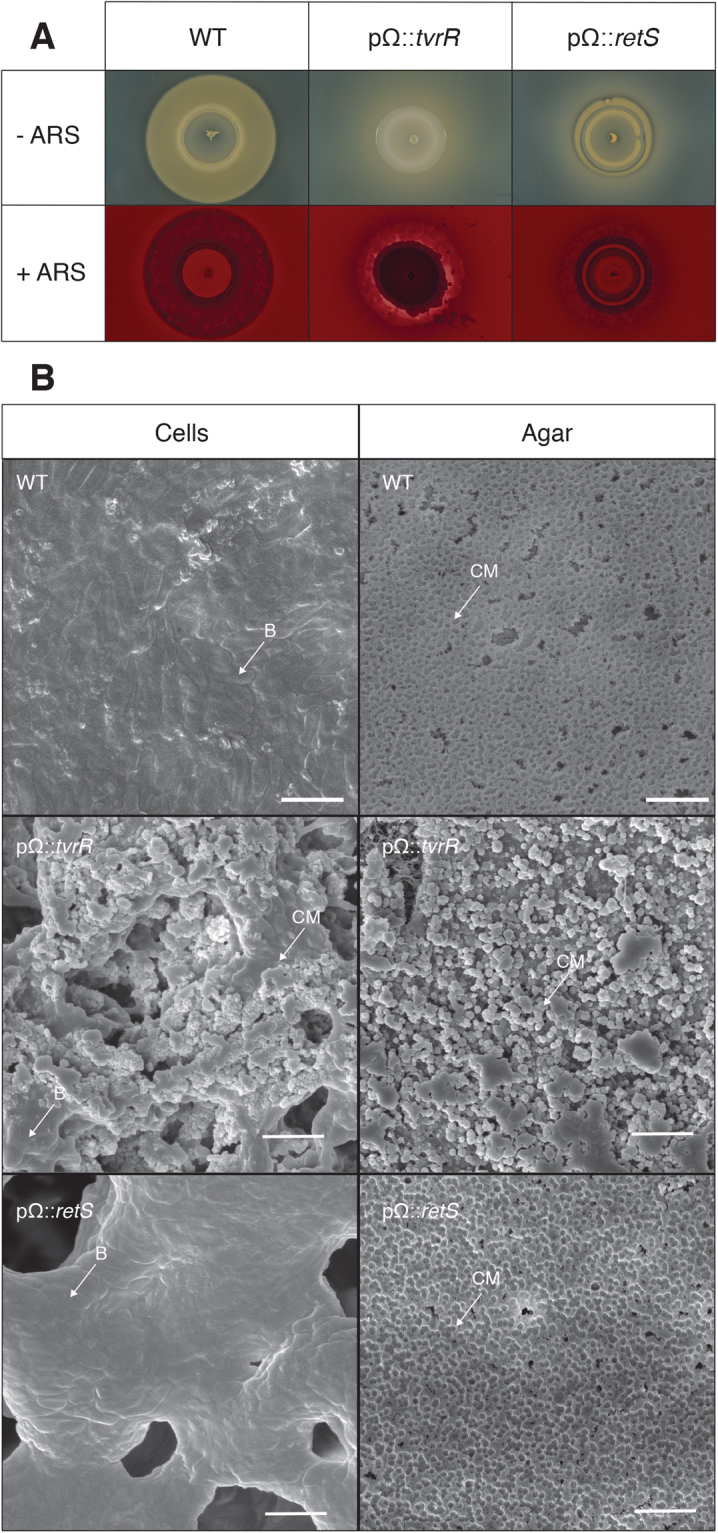
(**A**) Pictures of *P. syringae* pv. tomato DC3000, *P. syringae* pv. tomato DC3000 pΩ::*tvrR*, and *P. syringae* pv. tomato DC3000 pΩ::*retS* grown on NB agar supplemented with calcium after four days of growth on plates. Pictures were taken of the same colony before and after staining of the colonies with 1.0% (w/v) ARS. These pictures are representative of the phenotype observed in each strain after four days of growth on NB agar supplemented with calcium. This assay was repeated with three independent biological replicates and these pictures are representative of what was observed each time. (**B**) SEM images at 18,000x magnification of colony surface for *P. syringae* pv. tomato DC3000, agar adjacent to *P. syringae* pv. tomato DC3000, colony surface for *P. syringae* pv. tomato DC3000 pΩ::*tvrR*, agar adjacent to *P. syringae* pv. tomato DC3000 pΩ::*tvrR*, colony surface for *P. syringae* pv. tomato DC3000 pΩ::*retS*, and agar adjacent to *P. syringae* pv. tomato DC3000 pΩ::*retS* after six days of growth on NB supplemented with Ca^2+^. 2 µm scale bars are indicated in the lower right-hand corner of the images. An arrow pointing to calcium phosphate (CM) and an arrow pointing at bacteria (**B**) are included in the images when calcium phosphate or bacteria were present. Bacterial cells and agar were imaged with three independent biological replicates. The images used are representative of what was observed each time.

Surprisingly, the global regulators TvrR and RetS appeared to be involved in regulating calcium precipitation in *Pto*. Disruption of *tvrR* resulted in altered calcium precipitation, while disruption of *retS* resulted in little to no amorphous apatite precipitation in the form of a brown halo around the colony. Both of these genes are known as global regulators of virulence in *P*. *syringae* and we therefore further characterized the calcium precipitate defect in *tvrR* and *retS* mutant strains (pΩ::*tvrR* and pΩ::*retS*)^34,35^. The phenotypes of the *tvrR* and *retS* mutant strains were consistent with the Tn mutants (Tn5-38 and Tn5-59) and did not appear different than WT *Pto* when grown on NB agar without supplemental Ca^2+^ added (Figs 7, S11). The phenotype of the pΩ::*tvrR* mutant when grown on NB supplemented with Ca^2+^ showed that the surface of the strain stained with ARS (Fig. 7A). This suggests that the opaque nature of this strain could be due to calcium precipitation on the colony surface. SEM of a pΩ::*tvrR* colony six days after spotting on NB supplemented with Ca^2+^ confirmed that calcium phosphate was accumulating on the surface of the colony (Fig. 7B). In addition, calcium phosphate precipitate in the agar adjacent to the pΩ::*tvrR* cells was similar in morphology to the calcium phosphate particles found in the agar next to *P. syringae* pv. morsprunorum 5795 at 6 days of growth (Fig. 7B). The pΩ::*retS* strain was not stained by ARS on the surface of the colony and very little of the agar surrounding the strain was stained (Fig. 7A). SEM images of the pΩ::*retS* strain showed that there was no calcium precipitate on the cell surface. However, even though the phenotype of the pΩ::*retS* strain looked distinct compared to WT *Pto*, the agar adjacent to the cells had a precipitate with a morphology reminiscent of the amorphous apatite found in the agar adjacent to WT *Pto* (Fig. 7B). Deletion of *retS* in *P. syringae* pv. syringae B728a results in a mucoidy phenotype due to the overproduction of alginate^35^. We compared the phenotype of the alginate non-producing ∆*algD Pto* strain to WT when grown on NB supplemented with Ca^2+^. No difference in the calcium precipitation phenotype were observed between the ∆*algD* strain and WT (Fig. S12), suggesting that alginate is dispensable for amorphous apatite formation. Overall, we confirmed that TvrR and RetS are involved in regulation of calcium precipitation in *Pto*.

## Discussion

Here we report the ability of 14 different *Pseudomonas* strains to precipitate amorphous calcium phosphate or amorphous apatite when grown on NB agar supplemented with Ca^2+^. We then identified several genes involved in calcium precipitation in *Pto*. Many of the genes encode for proteins with metabolic function, however approximately half of the genes have also been characterized as virulence genes. We further confirmed that the regulators TvrR and RetS regulate calcium phosphate precipitation in *Pto*.

Calcium precipitation was first assayed using ARS to stain calcium rich areas on the colonies and the agar around where the colonies grew. While this proved to be a quick and simple assay to test for the spatial distribution and the possibility of calcium precipitation, it also had several drawbacks. This includes the fact that areas stained by ARS represent calcium rich areas that may not have any calcium precipitates present, since ARS stains calcium indiscriminately^36^. Data obtained using Raman spectroscopy and SEM showed that ARS stained areas commonly represented areas where amorphous calcium phosphate or amorphous apatite was present. Interestingly, calcium carbonate was not precipitated by any of the *Pseudomonas* species assayed. Several *Pseudomonas* species have previously been shown to precipitate calcium carbonate under different conditions^16,17,37,38^. It is possible that the conditions we used to grow the *Pseudomonas* species facilitated the precipitation of calcium phosphate over calcium carbonate.

During MICP, bacterial cells act as nucleation points for calcium precipitation on the surface of bacterial colonies. Bacteria like *E. coli*, *Bacillus subtilus*, *C. matruchotii*, and *Chromohalobacter marismortui* all precipitate calcium on the surface of cells^4,10,39,40^. However, calcium phosphate precipitated only on the colonies of seven of the *Pseudomonas* strains assayed. There was no set pattern to the surface-associated calcium precipitation phenotype among the strains assayed since strains within the same *Pseudomonas* species or strains that had similar lifestyles differed in the development of surface-associated calcium precipitation (Table 1). EPSs are thought to be a common nucleation point during MICP, however we did not detect alginate or cellulose on the surface of any *Pseudomonas* colonies assayed. It has recently been observed in *Bacillus* sp. JH7 and *P. aeruginosa* that EPS production does not always correlate with bacterial calcium precipitation^41,42^. Our findings suggest a similar trend. MICP is also thought to be associated with an increase in pH as calcium precipitation is more likely to spontaneously and rapidly occur at a higher pH^1,43–45^. While all the *Pseudomonas* strains assayed raised the pH of the surrounding media, the colonies for several *Pseudomonas* strains that precipitated calcium phosphate on the surface of cells became more acidic after two days of growth. MICP under acidic conditions would seem counterintuitive but appeared to occur in several *Pseudomonas* strains.

**Table 1:** *Pseudomonas* species and strains used in this work.

*Pseudomonas* species	Origin of isolation	Properties^a^	Calcium precipitation on cell surface	Calcium precipitation in agar/(color)	Reference
*Pseudomonas putida* KT2440	Soil	Plant beneficial microbe	No	Yes/white	^56^
*Pseudomonas fluorescens* Pf0-1	Rif resistant mutant generated from a soil-borne isolate of *P. fluorescens*	Plant beneficial microbe	Yes	Yes/white	^57^
*Pseudomonas fluorescens* 55	Soil	Plant beneficial microbe	Yes	Yes/white	^58^
*Pseudomonas fluorescens* A506	Foliar	Plant beneficial microbe	Yes	Yes/white	^59^
*Pseudomonas fluorescens* SBW25	Soil	Plant beneficial microbe	No	Yes/white	^60^
*Pseudomonas* spp. 92	Phyllosphere	Plant beneficial microbe	No	Yes/brown	^61^
*Pseudomonas savastanoi* pv. savastanoi 4352	Foliar	Plant pathogen	Yes	Yes/brown	A. Collmer
*Pseudomonas syringae* pv. tomato DC3000	Foliar	Plant pathogen	No	Yes/brown	^62^
*Pseudomonas syringae* pv. glycinea 2159 Race 1	Foliar	Plant pathogen	No	Yes/brown	A. Collmer
*Pseudomonas syringae* pv. morsprunorum 5795	Foliar	Plant pathogen	Yes	Yes/white	A. Collmer
*Pseudomonas syringae* pv. maculicola ES4326	Foliar	Plant pathogen	Yes	Yes/brown	^63^
*Pseudomonas syringae* pv. tabaci ATCC11528	Foliar	Plant pathogen	Yes	Yes/brown	A. Collmer
*Pseudomonas syringae* pv. syringae B728a	Foliar	Plant pathogen	No	Yes/brown	^64^
*Pseudomonas viridiflava* NYS-1	Foliar	Plant pathogen	No	Yes/brown	A. Collmer

^a^In reference to the main life-style of the bacteria in relation to how it associates with plants.

All the *Pseudomonas* strains assayed precipitated calcium phosphate as a white halo in the agar adjacent to the colonies. The calcium phosphate that precipitated in the white halo was amorphous, non-crystalline, and was likely precipitated through MICP^8^. Among the *Pseudomonas* strains assayed, six of the seven *P. syringae* pathovars and *P*. spp. 92 precipitated a brown halo of amorphous apatite around the colonies. In the case of all the *Pseudomonas* where this occurred, the brown halo replaced a white halo that had been in the same location on the agar plate. This suggests that the *Pseudomonas* strains actively facilitate formation of this brown halo of amorphous apatite. Active formation of apatite is characteristic of MCCP^7^. However, a couple characteristics of this amorphous apatite make it unlikely to be produced through MCCP. First, the morphology of the amorphous apatite was unordered and fibrous. Although these fibers displayed similarities with artificial bone precursors, they did not have an ordered crystalline structure expected during MCCP^7,46^. Second, bacteria produce an organic matrix during MCCP and our analyses did not identify anything that could be considered a bacterially produced organic matrix. As such, this apatite would likely be characterized as MICP even though it had several interesting characteristics. Further characterization of genes involved in this apatite formation could help determine the mechanism of its formation.

Biogenic apatite has applications in medical and conservation fields. *P. fluorescens* SBW25 produces apatite when grown on concrete in lysogeny broth (LB) supplemented with phosphate and could be used for preservation purposes^20^. Although we did not perform our assays in LB supplemented with phosphate, we found that *P. fluorescens* SBW25 precipitated less calcium phosphate than other *P. fluorescens* strains assayed. Our results suggest that *P. fluorescens* strains, like *P. fluorescens* Pf0-1 or *P. fluorescens* 55, may be desirable *P. fluorescens* strains to use if biogenic apatite is needed for application purposes. Our results may aid in choosing or applying the appropriate *Pseudomonas* species for a particular application involving biogenic apatite.

Our Tn-mutant screen identified several global regulators and metabolic pathways involved in regulating calcium phosphate precipitation during growth of *Pto*. The Tn-mutant with an insertion in the gene *phoU* was one of the mutants that did not precipitate amorphous apatite in the agar adjacent to the colony. In *P. aeruginosa*, *phoU* regulates phosphate metabolism and deletion of *phoU* results in accumulation of ppGpp and polyphosphate in cells^47^. Secretion of orthophosphate from stored polyphosphate is reported to be involved in MICP^18^. The fact that the *phoU* mutant shows reduced and altered calcium precipitation suggests a link between polyphosphate accumulation and calcium precipitation in *Pto*. Further analysis of the link between *phoU* and calcium phosphate precipitation could provide a greater understanding into the role polyphosphates and phosphate metabolism play in this phenomenon.

Further analysis of the Tn-mutants showed that several genes linked to calcium precipitation, eight out of the 17 identified, have been assigned direct or indirect roles in virulence of *P*. *syringae*, another bacterial plant pathogen, or a related pathogenic *Pseudomonas* species (Table S2). Notably, the global regulators of virulence in *P*. *syringae*, *retS*, *tvrR*, and *cbrB*, were among the genes linked to calcium precipitation^34,35,48^. Ca^2+^ is abundant within the leaf apoplast and is an important secondary messenger during the plant defense response^24,49^. Some bacterial plant pathogens chelate Ca^2+^ *in planta* with EPSs to reduce the defense response^50^. Both alginate and cellulose chelate Ca^2+^ but are disposable during *Pto* virulence^51,52^. As such, there could be another mechanism through which *Pto* disables Ca^2+^ signaling *in planta* during infection. As calcium precipitation seems to correlate with virulence related genes, investigation into whether *Pto* induces calcium precipitation to occur *in planta* could be worthwhile. If *Pto* does induce calcium precipitation *in planta*, it could be a novel mechanism that plant pathogens use to disrupt the plant immune response. A more thorough characterization of TvrR, RetS, and CbrB could identify the mechanism through which *Pto* regulates calcium phosphate precipitation and help determine whether calcium precipitation directly relates to virulence in *P*. *syringae*.

## Materials and Methods

### Growth and cultivation of *Pseudomonas* species

*Pseudomonas* (Table 1) were routinely cultivated on Kings B (KB) agar^53^. Prior to inoculation, each strain was grown in KB medium for 10–12 hours and then washed twice in Nutrient Broth (NB) (Becton, Dickinson, and Company, Franklin Lakes, NJ) medium before being resuspended into NB medium at an optical density of 0.3 measured at 600 nm (OD_600_). 5 µL of each bacterial suspension was then spotted onto individual NB agar plates and individual NB agar plates supplemented with 5 mM CaCl_2_. For controls, individual NB agar plates supplemented with 5 mM CaCl_2_ were spotted with 5 µL of autoclaved cells from each strain or left blank.

### pH measurement of *Pseudomonas* cultures

Cultures were grown to stationary phase in KB medium and then washed twice in NB medium before being resuspended in NB medium at an OD_600_ of 0.3. 5 µL of each bacterial suspension was spotted on an NB agar plate supplemented with 5 mM CaCl_2_ and 0.1% (w/v) BB and monitored for two days.

### Alizarin red S staining

The method of ARS staining of calcium rich areas was modified from histological methods for the purposes of staining agar plates and bacterial colonies^36^. NB agar plates and NB agar plates supplemented with 5 mM CaCl_2_ spotted with bacterial colonies were flooded with 1.0% (w/v) ARS (Sigma-Aldrich, St. Louis, MO), pH 4.1. After five minutes, the dye was removed using a pipette and plates were washed with 1 mL of ddH_2_O to remove excess ARS. The dark red areas were scored as calcium rich areas. ARS staining was repeated with five biological replicates for each strain.

### Raman spectroscopy

Strains grown on NB agar plates supplemented with 5 mM CaCl_2_ and uninoculated NB agar plates supplemented with 5 mM CaCl_2_, were directly used for analysis. Raman spectroscopy was performed using a Renishaw InVia Confocal Raman Microscope (Renishaw, Illinois, IL) with a 785 nm laser. Spectra were taken at the center of each bacterial colony and on the agar directly adjacent to bacterial colonies every day for six days.

### Scanning electron microscopy (SEM)

One or six-day old bacterial colonies grown on NB agar plates supplemented with 5 mM CaCl_2_ were frozen in nitrogen slush and freeze-dried. Freeze-dried bacterial colonies were removed from the agar and mounted directly on aluminum pegs using carbon tape. Subsequently, agar directly adjacent to the bacterial colonies was mounted on aluminum pegs using carbon tape. Mounted samples were then coated with gold-palladium in a Desk V sputter coater (Denton Vacuum, Moorestown, NJ). SEM was performed on a TESCAN Mira3 FESEM (Tescan, Czech Republic) using an In-Beam detector set at 5 kV.

### Transposon Mutagenesis

Mutations were made using the EZ-Tn5^TM^ <KAN-2> Tnp Transposome^TM^ Kit (Illumina, Madison, WI) following the manufacturer’s protocol. Briefly, *P. syringae* pv. tomato DC3000 electrocompetent cells were prepared as described previously^54^. For the mutagenesis reaction, 1 µl of EZ-Tn5^TM^ <KAN-2> Tnp Transposome^TM^ was added to 100 µl of electrocompetent *P*. *syringae* pv. tomato DC3000 cells. Cells were immediately electroporated using the Bio-Rad (Hercules, CA) Gene Pulser XCell^TM^ electroporation system with the following settings: 2 mm electroporation cuvette; 2.5 kV; 25 µF; 200 Ω. Cells were immediately recovered in 1 ml of LM medium and incubated at 28 °C for 3 hours^55^. After recovery, the cell suspension was diluted 1:12 in LM medium and 100 µl aliquots were plated onto a total of 120 NB agar plates supplemented with 50 µg/ml of kanamycin and 5 mM CaCl_2_. Plates were incubated at room temperature for 7 days and monitored for growth of colonies and calcium precipitation. Colonies that either had little or no visible halo of brown precipitate, colonies with white precipitate on the colony surface, and colonies with a halo of brown precipitate greater than WT were selected for further analysis.

### Identification of transposon insertion site

To identify the insertion site each transposon (Tn) mutant, was grown overnight in KB supplemented with 50 µg/mL kanamycin at 28 °C. Overnight cultures were used to extract genomic DNA using the Wizard® Genomic DNA Purification Kit (Promega, Madison, WI). Genomic DNA samples from each of the Tn5 mutant strains were used as templates for arbitrary PCR reactions. For each strain, two rounds of PCR were performed. Primers used to identify Tn insertions are found in Table S3. Round 1 used one primer specific to the Tn sequence (oSWC02330 for amplifying sequences adjacent to the 5′ end of the Tn sequence, and oSWC01139 for amplifying sequences adjacent to the 3′ end of the Tn) and one primer (oSWC0141) that contains the 5 bp sequence, GAACG, that is found to occur randomly at approximately every 400 nt in the *P. syringae* pv. tomato DC3000 genome and a specific tail sequence that is used in the second round of PCR reactions. Round 2 PCR reactions were carried out using a nested primer that is contained within the round 1 PCR amplicon and specific to the Tn sequence, but does not overlap the primer sequence used in round 1 (oSWC02332 5′ end; oSWC02331 3′ end) and a primer specific to the tail sequence of the oSWC0141 primer used in round 1 (oSWC0142). This allows for specific amplification of sequences immediately adjacent to the ends of the Tn for each mutant strain, and thus identification of the site of insertion. Tn insertion sites were considered correct only when sequencing results from both ends of the Tn could be mapped to the same locus in the genome of *P*. *syringae* pv. tomato DC3000. All PCR reactions were 25 µl. Round 1 PCR reactions contained: 1 µl gDNA (~500 ng); 12.5 µl OneTaq® 2x Master Mix (New England Biolabs Inc., Ipswich, MA); 2 µM of each primer; H_2_O to 25 µl. Thermocycling conditions: 94 °C, 30 s; (94 °C, 30 s; 42 °C, 30 s; 68 °C, 3 min) for 6 cycles; (94 °C, 30 s; 52 °C, 30 s; 68 °C, 3 min) for 25 cycles; 68 °C for 7 minutes. After round 1 PCR, the reactions were cleaned to remove excess primers and gDNA using the QIAquick PCR Purification Kit (QIAGEN, Valencia, CA) following the manufacturer’s protocol. Round 2 PCR reactions contained: 1.5 µl of purified PCR product from round 1 as template; 0.2 µM of oSWC0142 primer and 2 µM of 2^nd^ round nested primer (oSWC02332 or oSWC02331); H_2_O to 25 µl. Thermocycling conditions were similar to round 1 PCRs with the exception that a touchdown PCR protocol was used where the annealing step started at a temperature of 63 °C and decreased by 1 °C each cycle for 13 cycles, and then remained at 50 °C for an additional 17 cycles. After round 2 PCR, samples were cleaned using ExoSAP-ITTM PCR Product Cleanup Reagent (Thermo Fisher Scientific, Waltham, MA) as per manufacturer’s instructions. Cleaned PCR products were sequenced at the Cornell University Biotechnology Resource Center using an Applied Biosystems Automated 3730xl DNA Analyzer using sequencing primer oSWC2209 for 3′ end products, and oSWC2210 for 5′ end products.

## Electronic supplementary material

Supplemental information
