# Supplementary material for: Physiological and genetic characterization of calcium phosphate precipitation by Pseudomonas species
Source: Sci Rep. 2018 Jul 5;8:10156. doi: 10.1038/s41598-018-28525-4 (PMC6033914; doi:10.1038/s41598-018-28525-4)
Supplement: Supplementary file 1 — Supplemental information [file 41598_2018_28525_MOESM1_ESM.pdf]

Supplemental Information

Physiological and genetic characterization of calcium phosphate precipitation by *Pseudomonas* species

Maxwell R. Fishman[1], Krista Giglio[2], David Fay[1], and Melanie J. Filiatrault[1,2,*]

[1]School of Integrated Plant Science, Section of Plant Pathology and Plant-Microbe Biology, Cornell University, Ithaca, NY 14853
[2] Emerging Pests & Pathogens Research Unit, Robert W. Holley Center, USDA-Agricultural Research Service, Ithaca NY 14853

*Corresponding Author:
Email: Melanie.filiatrault@ars.usda.gov

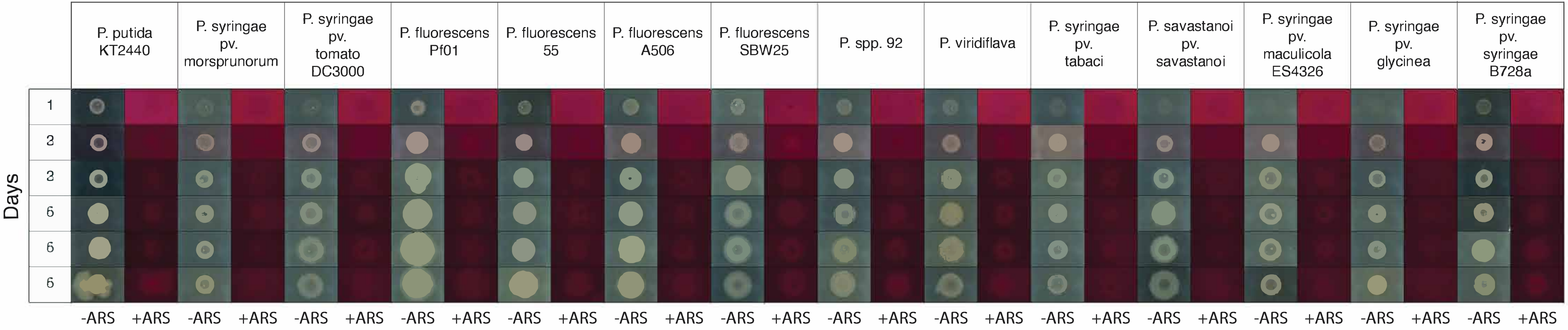

Fig. S1: Pictures of Pseudomonas species grown on NB agar over the course of six days. Pictures were taken of the same colony before (-) and after (+) staining of the colonies with 1.0% (w/v) ARS. These pictures are representative of growth for each Pseudomonas species on NB agar. The assay was repeated with three independent biological replicates.

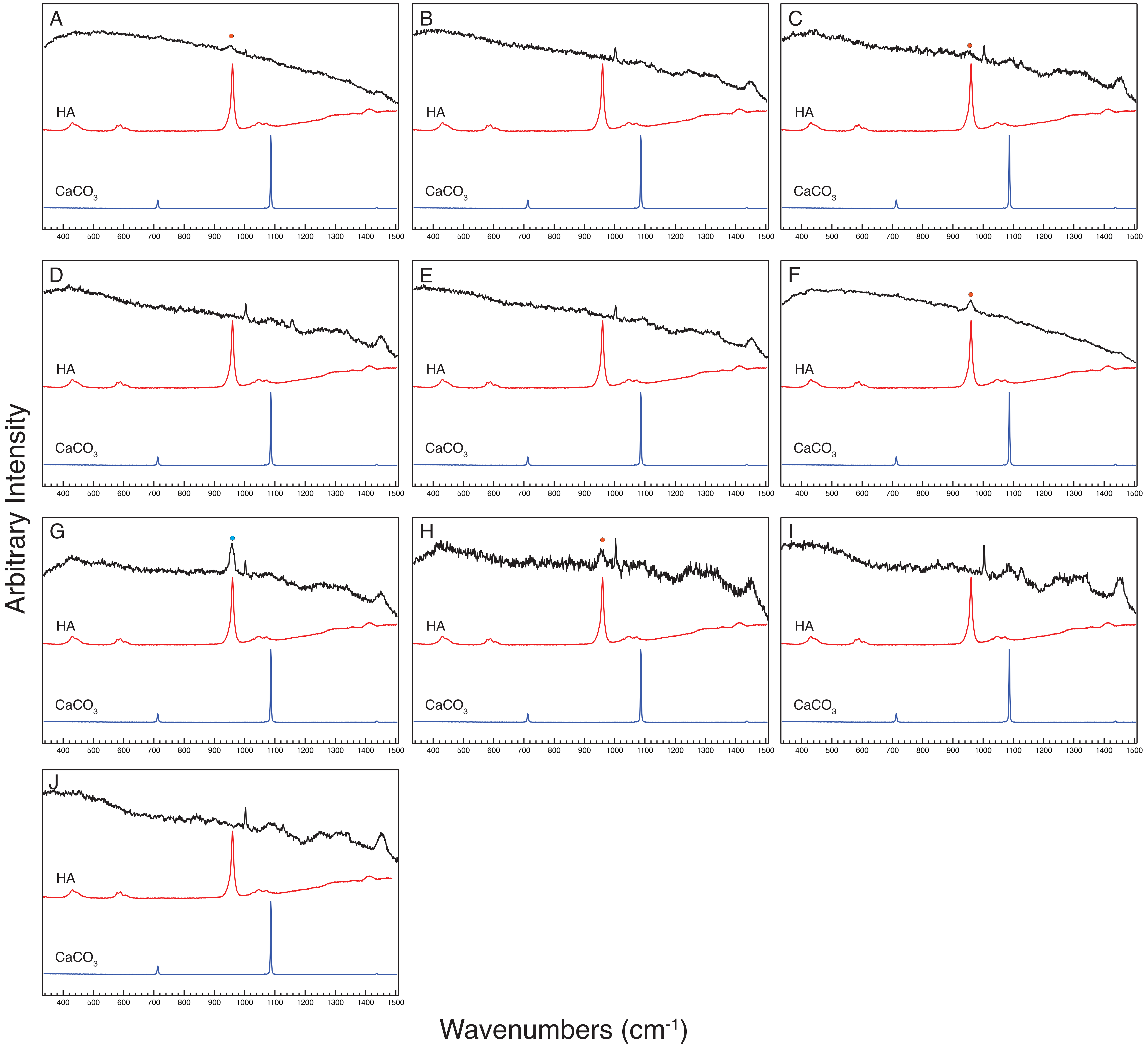

Fig. S2: Raman spectra of (A)*P. fluorescens* 55, (B) *P. fluorescens* SBW25, (C) *P. fluorescens* A506, (D) *P. viridiflava* NYS-1, (E) *P.* spp 92, (F) *P. syringae* pv. tabaci ATCC11528, (G) *P. savastanoi* pv. savastanoi 4325, (H) *P. syringae* pv. maculicola ES4326, (I) *P. syringae* pv. glycinea 2159 Race 1, and (J) *P. syringae* pv. syringae B728a colonies taken from 335 cm^{-1} to 1515 cm^{-1} after six days of growth on NB supplemented with Ca^{2+} in black. A hydroxyapatite control spectrum labelled "HA" that is colored red and a calcium carbonate control spectrum labelled "$CaCO_3$" that is colored blue is present each panel. A broad band in the bacterial colony spectra centered around 955 cm^{-1} is labelled with an orange dot where present and signifies the presence of amorphous calcium phosphate on the colony surface. A sharp band in the bacterial colony spectra centered at 959 cm^{-1} signifies the presence of amorphous apatite and is labelled with a light blue dot where present. Bands commonly associated with biological organisms, including a peak for DNA (782 cm^{-1}) and phenylalanine (1004 cm^{-1}) and broad bands associated with amides (1230-1300 cm^{-1}) and methyl groups (1430-1460 cm^{-1}) can be seen on these spectra as well. These spectra are representative of spectra taken from three independant biological replicates grown for the same amount of time under the same conditions.

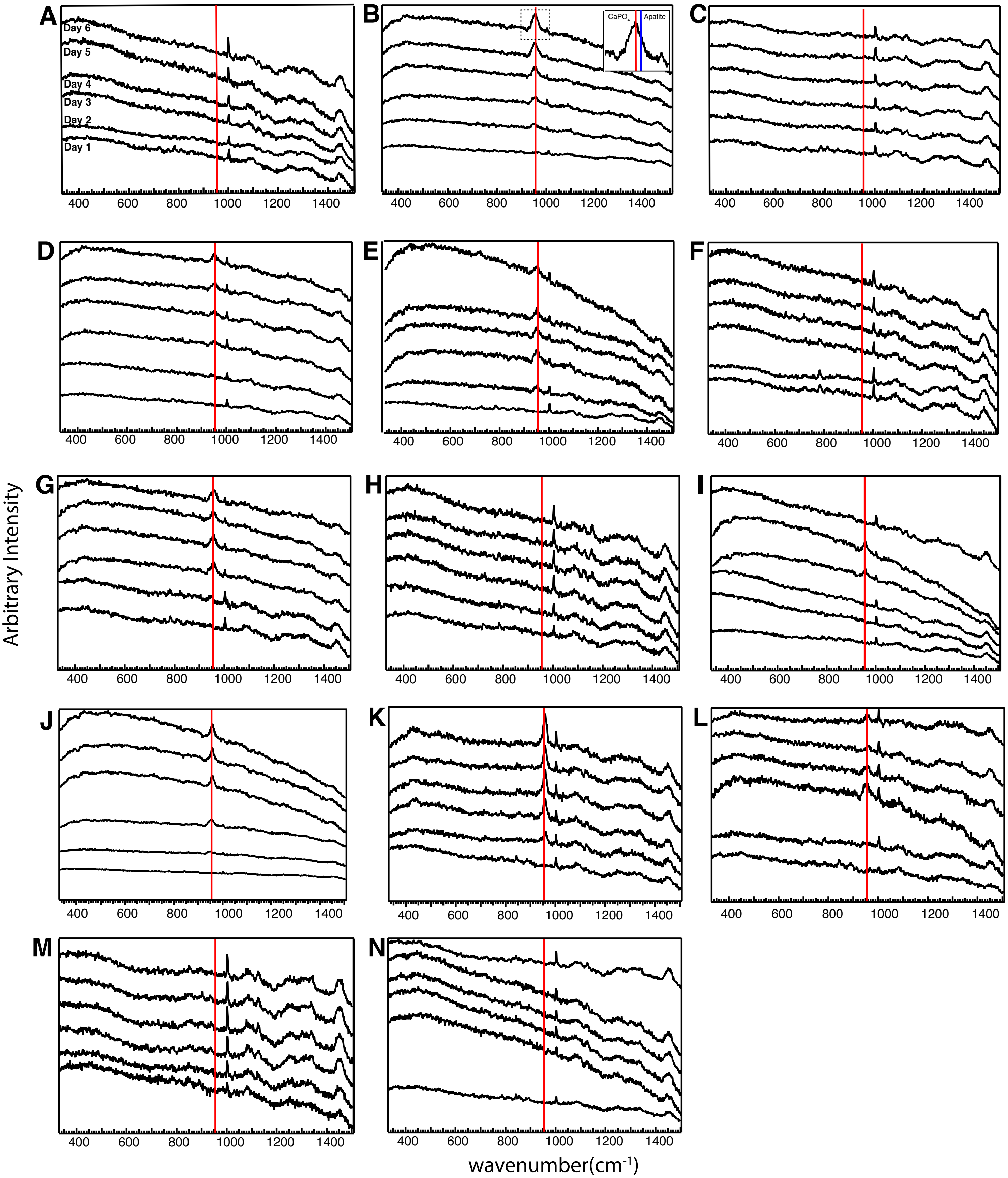

Figure S3: Raman spectra taken from the center of the surface of colonies of (A) *P. putida* KT2440, (B) *P. syringae* pv. morsprunorum 5795, (C) *P. syringae* pv. tomato DC3000, (D) *P. fluorescens* Pf0-1, (E) *P. fluorescens* 55, (F) *P. fluorescens* SBW25, (G) *P. fluorescens* A506, (H) *P. viridiflava* NYS-1, (I) *P.* spp. 92, (J) *P. syringae* pv. tabaci ATCC11528 (K) *P. savastanoi* pv. savastanoi 4325 (L) *P. syringae* pv. maculicola ES4326, (M) *P. syringae* pv. glycinea 2159 Race 1, and (N) *P. syringae* pv. syringae B728a at 1, 2, 3, 4, 5, and 6 days of growth as labeled in 2A. The y-axis is in arbitrary intensity units and the x-axis is in wavenumbers (cm^{-1}). The red line at 955 cm^{-1} indicates the center of a peak for amorphous calcium. The inset in panel 2B shows a close-up of the peak formed due to amorphous calcium phosphate production on the surface of *P. syringae* pv. morsprunorum 5795 colonies at six days of growth. The specific wavenumber where peaks for amorphous calcium phosphate (955 cm^{-1}) and apatite (961 cm^{-1}) occur are labeled in red and blue, respectively.

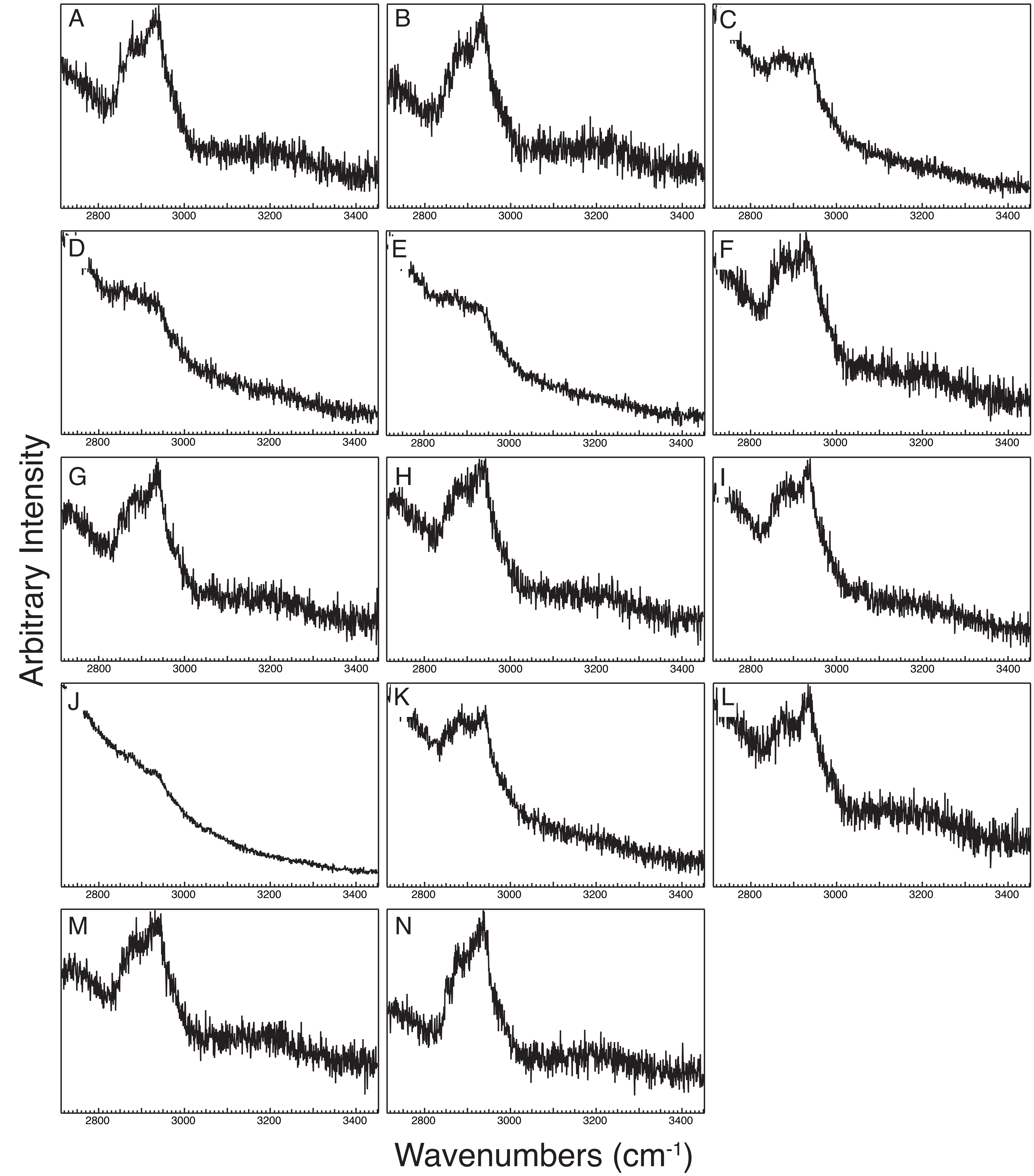

Fig. S4: Raman spectra of (A) *P. putida* KT2440, (B) *P. syringae* pv. tomato DC3000, (C) *P. fluorescens* Pf0-1, (D) *P. syringae* pv. morsprunorum 5795, (E) *P. fluorescens* 55, (F) *P. fluorescens* SBW25, (G) *P. fluorescens* A506, (H) *P. viridiflava* NYS-1, (I) *P.* spp 92, (J) *P. syringae* pv. tabaci ATCC11528, (K) *P. savastanoi* pv. savastanoi 4325, (L) *P. syringae* pv. maculicola ES4326, (M) *P. syringae* pv. glycinea 2159 Race 1, and (N) *P. syringae* pv. syringae B728a colonies taken from 2715 cm^{-1} to 3450 cm^{-1} after six days of growth on NB supplemented with Ca^{2+}. The peaks found centered at 2880 cm^{-1} and 2930 cm^{-1} reflect that lipids are present on the bacterial cells. These spectra are repesentative of spectra taken from three independent biological replicates that were grown for the same amount of time under the same conditions.

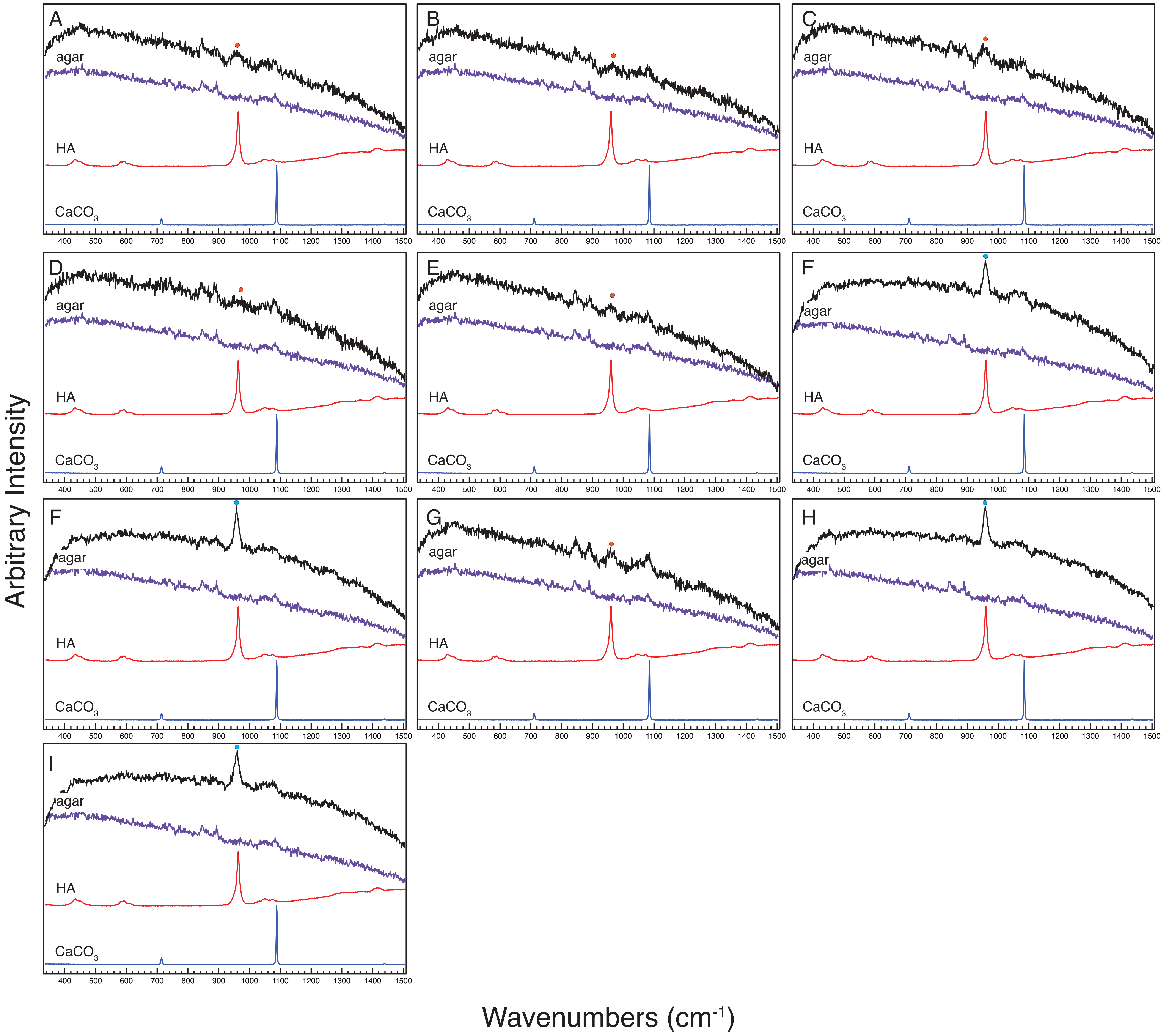

Fig. S5: Raman spectra of the agar adjacent to (A)*P. fluorescens* 55, (B) *P. fluorescens* SBW25, (C) *P. fluorescens* A506, (D) *P. viridiflava* NYS-1, (E) *P.* spp 92, (F) *P. syringae* pv. tabaci ATCC11528, (G) *P. savastanoi* pv. savastanoi 4325, (H) *P. syringae* pv. maculicola ES4326, (I) *P. syringae* pv. glycinea 2159 Race 1, and (J) *P. syringae* pv. syringae B728a colonies taken from 335 cm^{-1} to 1515 cm^{-1} after six days of growth on NB supplemented with Ca^{2+} in black. An agar control spectrum labelled "agar" that is colored purple, a hydroxyapatite control spectrum labelled "HA" that is colored red, and a calcium carbonate control spectrum labelled "$CaCO_3$" that is colored blue is present each panel. A broad band in the bacterial colony spectra centered around 955 cm^{-1} is labelled with an orange dot where present and signifies the presence of amorphous calcium phosphate on the colony surface. A sharp band in the bacterial colony spectra centered at 959 cm^{-1} signifies the presence of amorphous apatite and is labelled with a light blue dot where present. These spectra are representative of spectra taken from three indepenent biological replicates grown for the same amount of time under the same conditions.

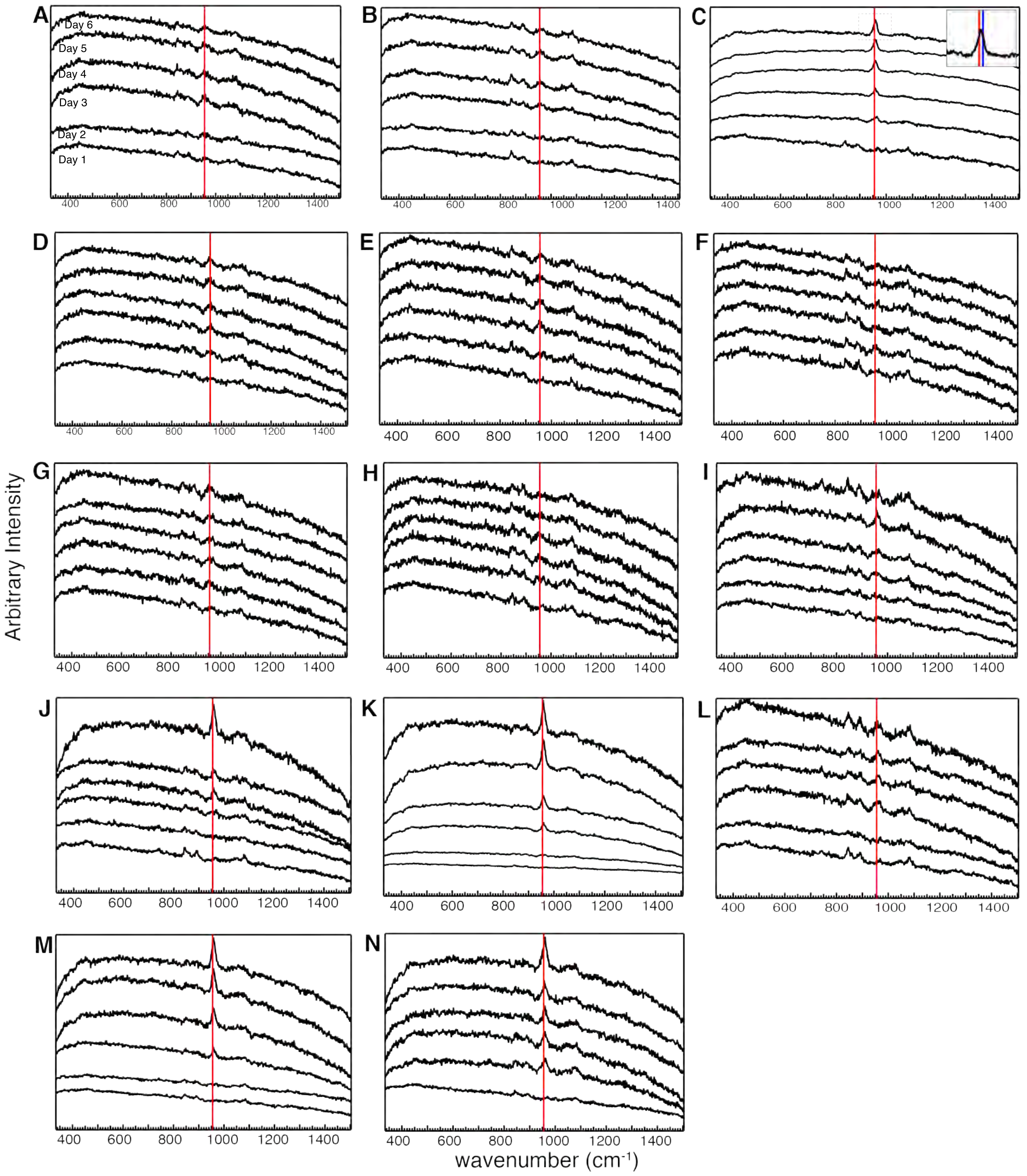

Figure S6: Raman spectra taken from the agar directly adjacent to live colonies of (A) *P. putida* KT2440, (B) *P. syringae* pv. morsprunorum 5795, (C) *P. syringae* pv. tomato DC3000, and (D) *P. fluorescens* Pf0-1, (E) *P. fluorescens* 55, (F) *P. fluorescens* SBW25, (G) *P. fluorescens* A506, (H) *P. viridiflava* NYS-1, (I) *P.* spp. 92, (J) *P. syringae* pv. tabaci ATCC11582 (K) *P. savastanoi* pv. savastanoi 4325 (L) *P. syringae* pv. *maculicola* ES4326, (M) *P. syringae* pv. glycinea 2159 Race 1, and (N) *P. syringae* pv. syringae B728a at 1, 2, 3, 4, 5, and 6 days of growth as labeled in 3A. The y-axis is in arbitraty intensity units and the x-axis is in wavenumbers (cm^{-1}). The red line is at 955 cm^{-1} indicates the center of a peak for amorphous calcium phosphate. The inset in panel 3C shows a close-up of the peak formed due to amorphous apatite production in the agar adjacent to a colony of of *P. syringae* pv. tomato DC3000 at six days of growth and the specific wavenumber where a peak for amorphous calcium phosphate (955 cm^{-1}) and apatite (961 cm^{-1}) would occur are labeled in red and blue, respectively. These spectra are representativeo of spectra taken from three independent biological replicats that were grown under the same conditions for the same amount of time.

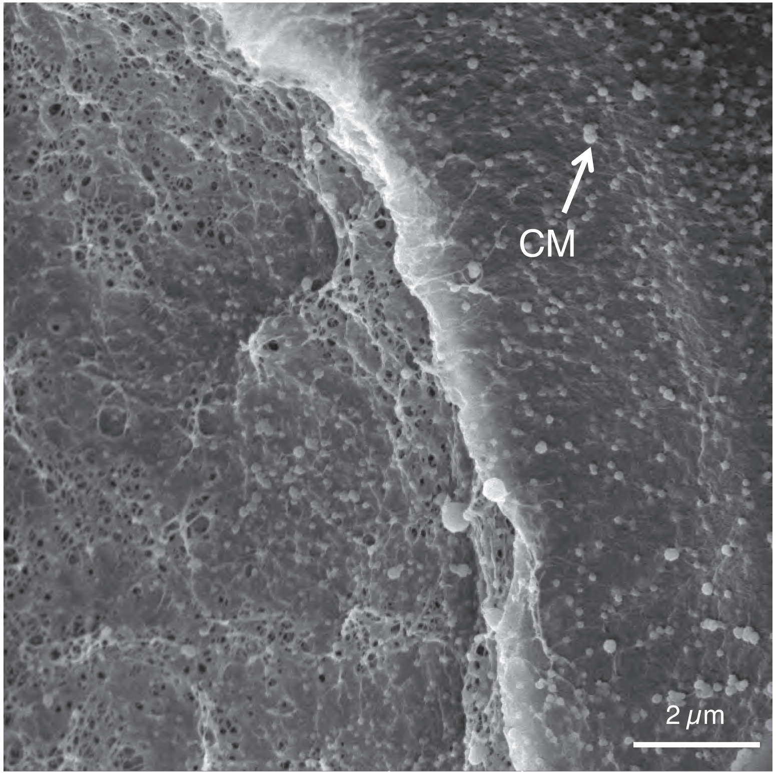

Figure S7: SEM images at (A) 18,000x and of NB agar with calcium. The arrow labeled "CM" indicates a calcium phosphate particle. The scale bar is indicated in the lower right hand corner of the image.

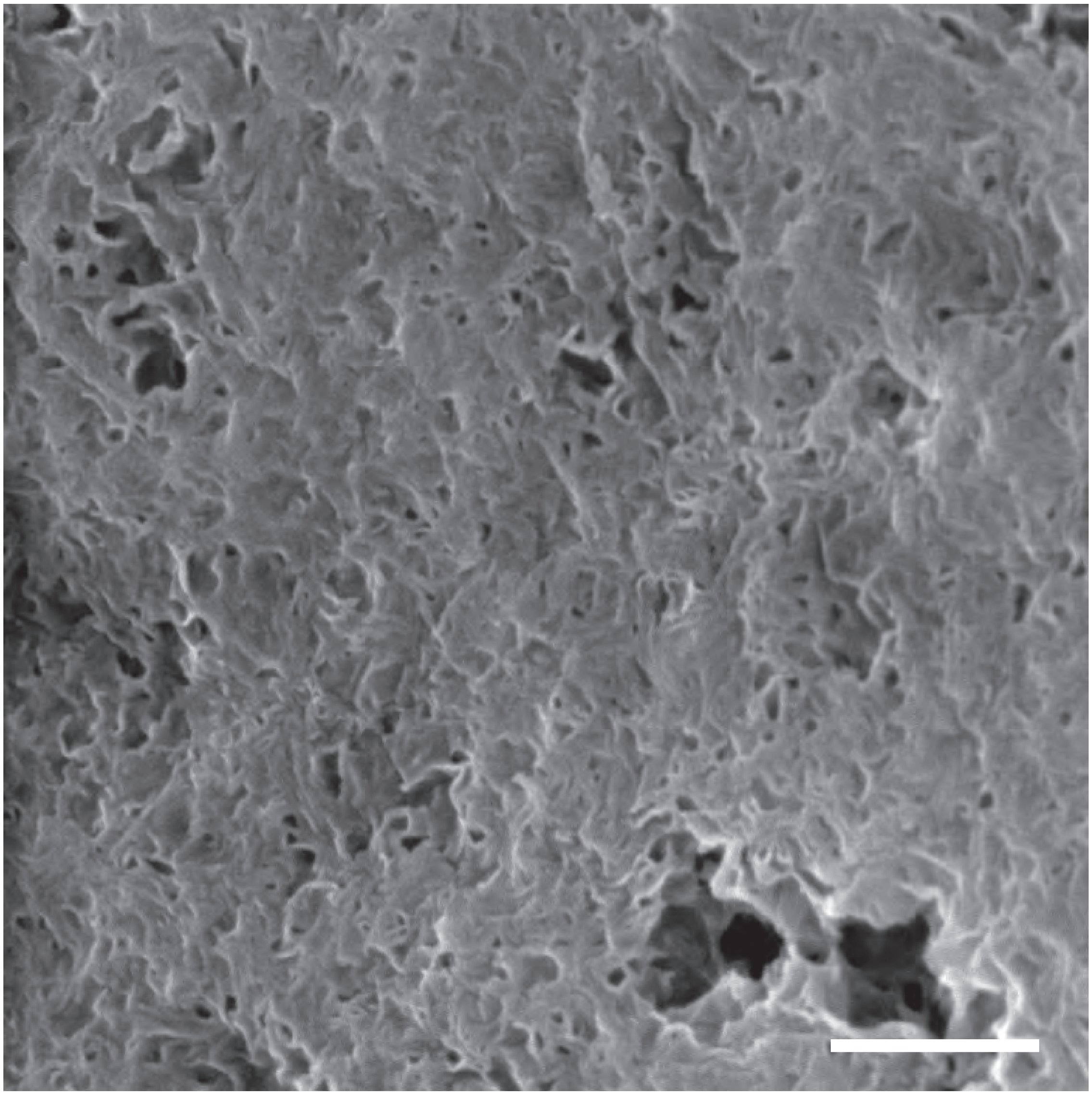

Figure S8: SEM image at 80,000x magnification of agar directly adjacent to *P. syringae* pv. tomato DC3000 after six days of growth. The entirety of the image is composed of the mineralized area of the agar. The scale bar is 500 nm and is indicated in the lower right hand corner of the image.

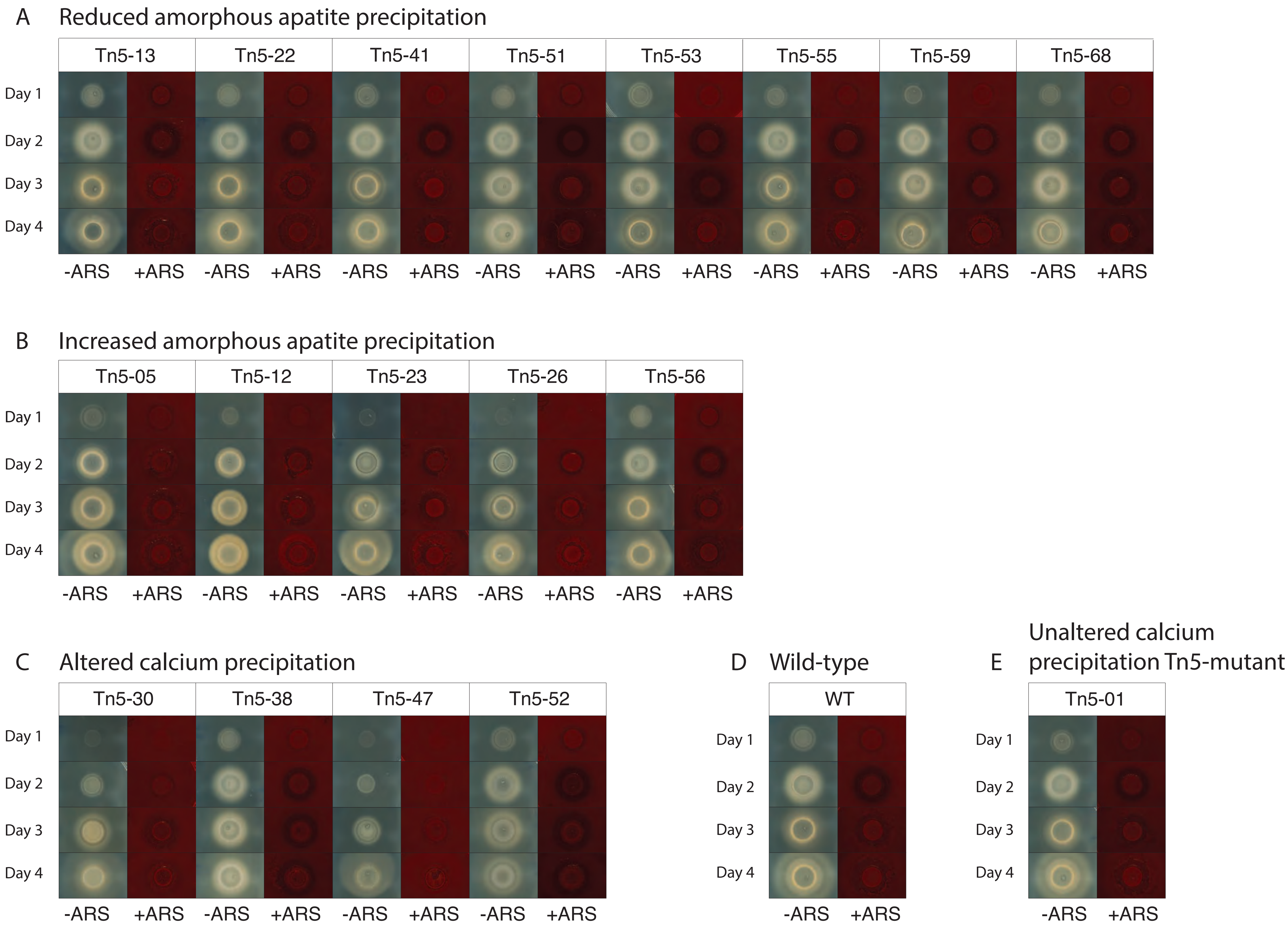

Fig. S9: Pictures of Tn5 mutants of *Pto* over the course of four days of growth that displayed diferences in calcium precipitation as compared to WT before and after ARS staining. Tn5 mutants are grouped according to phenotype during or after four days of growth. These phenotypes are either (A) reduced amorphous apatite precipitation, (B) increased amorphous apatite precipitation, and (C) altered calcium precipitation. These phenotypes were compared to (D) WT and considered (E) unaltered if they produced similar patterns of calcium precipitation after four days of growth as compared to WT. This assay was performed three indepent times and the photos are representative of the assays.

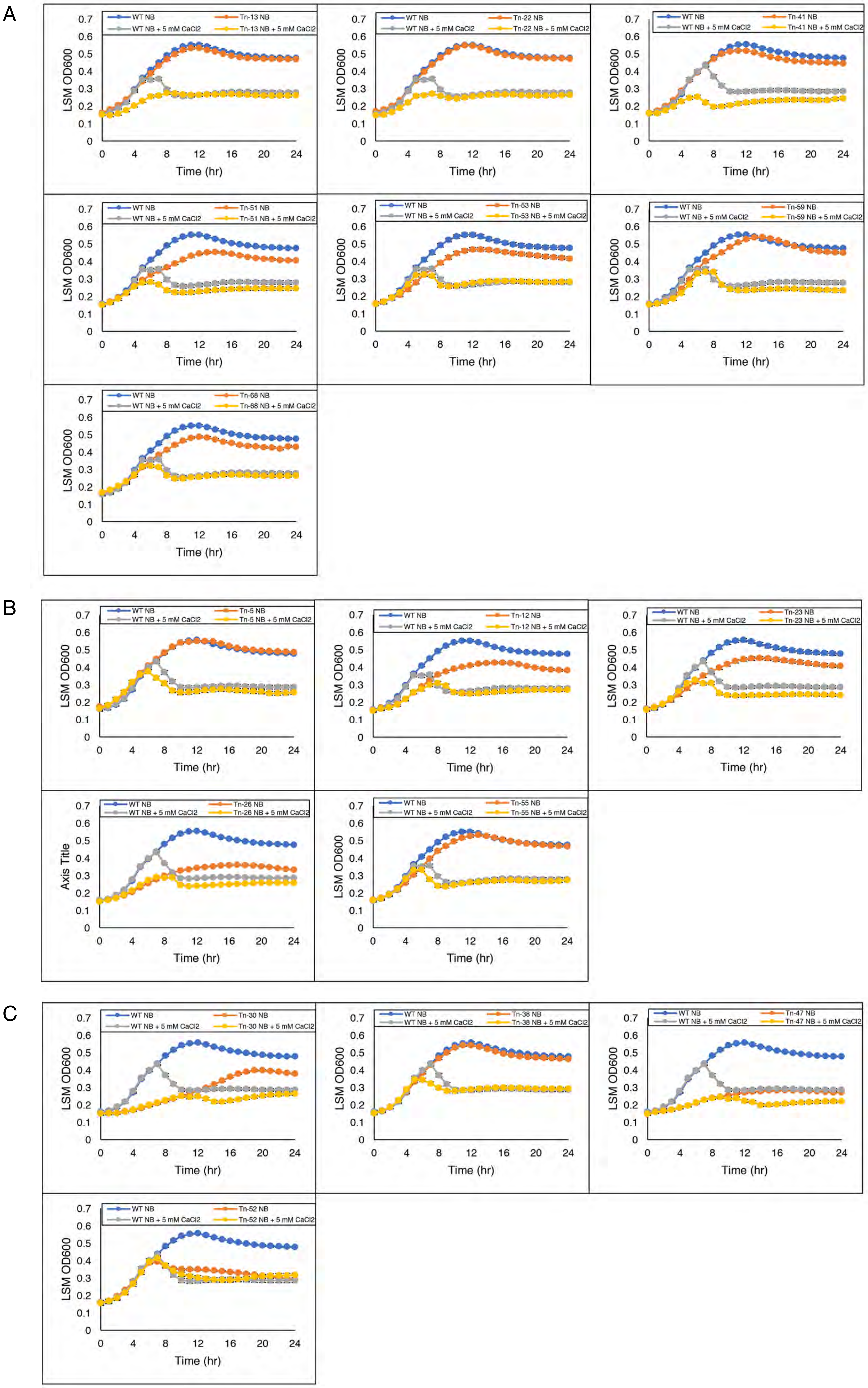

Fig. S10: Growth curves of Tn-mutants scored with (A) little to no, (B) excessive, or (C) altered calcium precipitation phenotypes as compared to WT when grown in NB medium or NB medium supplemented with 5 mM $CaCl_2$. The Tn-mutant being compared to WT in each panel can be found in the panel legend. Growth curves were made by compiling three independent experiments into a least-squares mean. Error bars represent standard error between experiments.

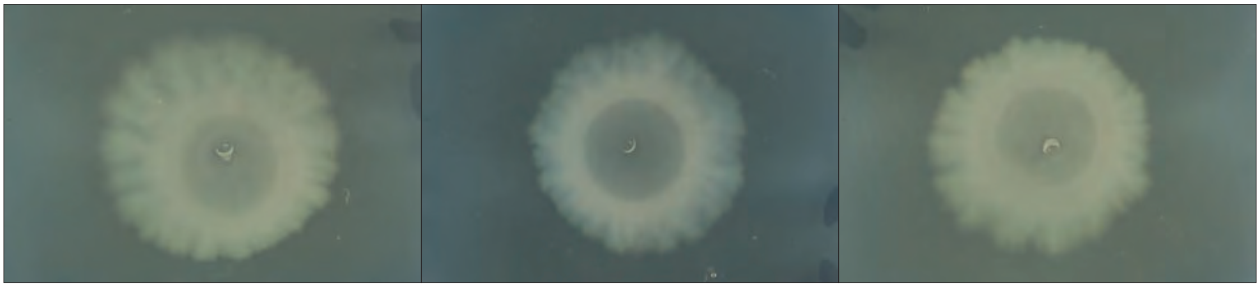

Pto *Pto* pΩ::*retS* *Pto* pΩ::*tvrR*

Fig. S11: Pictures of *Pseudomonas syringae* pv. tomato DC3000, *Pseudomonas syringae* pv. tomato DC3000 pΩ::*retS*, and *Pseudomonas syringae* pv. tomato DC3000 pΩ::*tvrR* after four days of growth on NB agar. These pictures are representative of phenotypes observed in these strains grown under these conditions during three independent biological replicates.

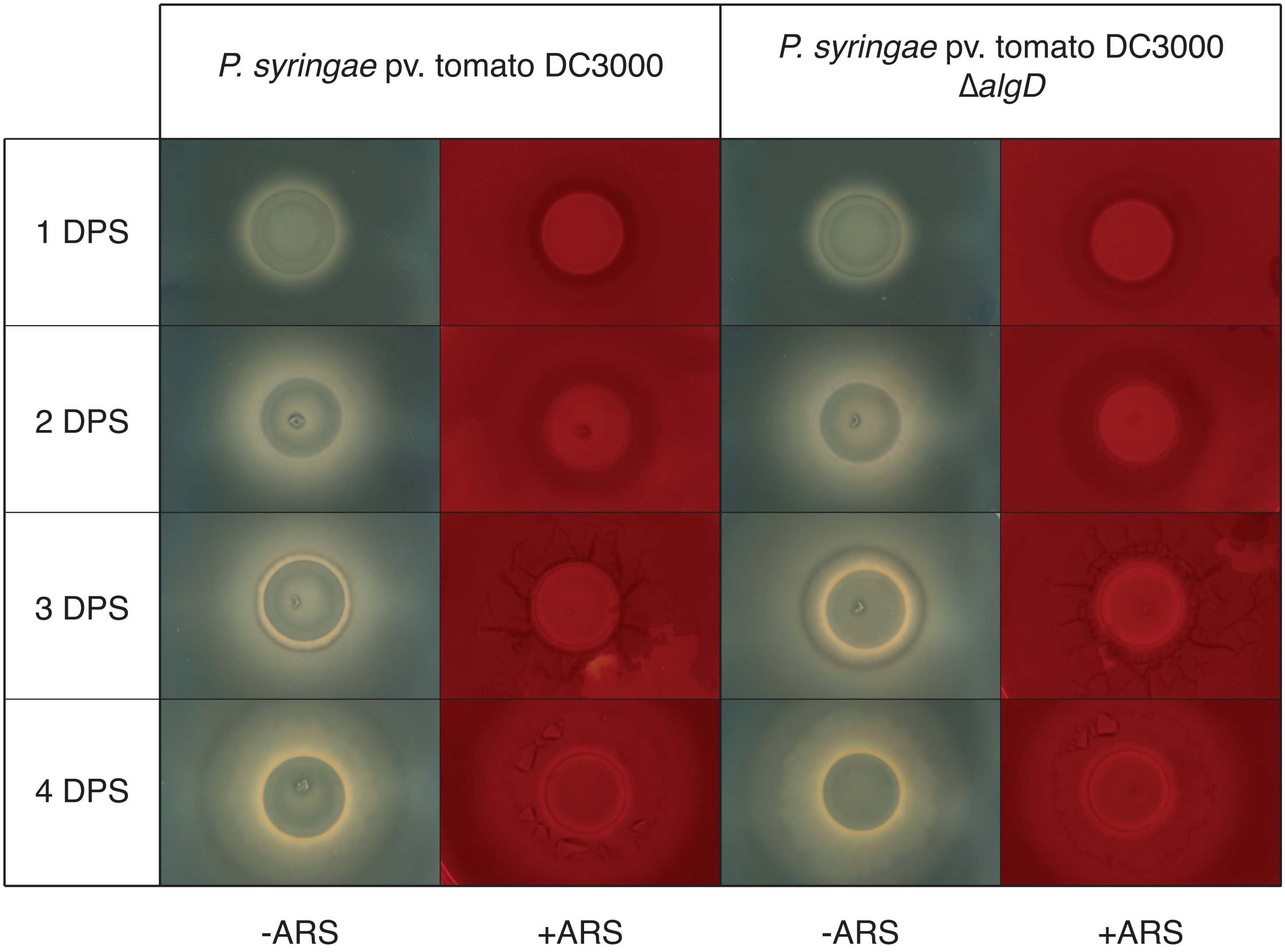

Fig. S12: Photos of WT and the Δ*algD* strain of *P. syringae* pv. tomato DC3000 when grown on NB supplemented with 5 mM $CaCl_2$. Photos were captured before and after ARS staining over the course of four days. This experiment was independently replicated three times and these photos are representative of the results observed for each replicate.

Table S1: Transposon mutant strains, observed phenotype, and the description of the disrupted gene

**Transposon Mutant Strain Number**	**Phenotype[a]**	**Gene Disrupted**	**Gene name**	**Protein Description[b]**	**Gene Ontology[c]**
Tn5-13	R	PSPTO_4986		Membrane protein, putative	
Tn5-22	R	PSPTO_1067		Glycosyl transferase, group 2 family protein	
Tn5-41	R	PSPTO_4991		Glycosyl transferase, group 1 family protein	
Tn5-51	R	PSPTO_2147		Pyoverdine sidechain peptide synthetase I, epsilon-Lys module	metabolic process [GO:0008152]
Tn5-53	R	PSPTO_5516	*uvrD*	DNA helicase (EC 3.6.4.12)	metabolic process [GO:0008152]
Tn5-55	R	PSPTO_0917	*ndh*	NADH dehydrogenase	oxidation-reduction process [GO:0055114]
Tn5-59	R	PSPTO_4868	*retS*	Sensor histidine kinase/response regulator RetS	
Tn5-68	R	PSPTO_3349	*ftsK*	DNA translocase FtsK	cell cycle [GO:0007049]; cell division [GO:0051301]; chromosome segregation

					[GO:0007059]
Tn5-05	E	PSPTO_3724	*lonI*	Lon protease	cellular response to stress [GO:0033554]; misfolded or incompletely synthesized protein catabolic process [GO:0006515]
Tn5-12	E	PSPTO_0965	*cbrB*	Sensor histidine kinase CbrB	regulation of transcription, DNA-templated [GO:0006355]; transmembrane transport [GO:0055085]
Tn5-23	E	PSPTO_0494	*bioH*	Biotin synthase	biotin biosynthetic process [GO:0009102]
Tn5-26	E	PSPTO_0496	*bioB*	BioH protein	
Tn5-56	E	PSPTO_1075		O-antigen ABC transporter, ATP-binding protein, putative	
Tn5-30	A	PSPTO_2194	*gltA*	Citrate synthase	tricarboxylic acid cycle [GO:0006099]

Tn5-38	A	PSPTO_3576	*tvrR*	TetR-like virulence regulator	regulation of transcription, DNA-templated [GO:0006355]; transcription, DNA-templated [GO:0006351]
Tn5-47	A	PSPTO_5006	*aceF*	Acetyltransferase component of pyruvate dehydrogenase complex (EC 2.3.1.12)	glycolytic process [GO:0006096]
Tn5-52	A	pSPTO_5483	*phoU*	Phosphate-specific transport system accessory protein PhoU	cellular phosphate ion homeostasis [GO:0030643]; negative regulation of phosphate metabolic process [GO:0045936]; phosphate ion transport [GO:0006817]

a. The phenotype observed during the confirmation of calcium preciptiation Tn-mutants. The phenotypes are as follows: A – altered calcium precipitation, E – excessive amorphous apatite precipitation, and R – reduced amorphous apatite precipitation
b. Description of protein coded for by the gene as found using Uniprot [3].

c. The gene ontology for the protein coded for each gene. This was collected using Uniprot [3].

Table S2: Genes found in Tn-mutant screen to be associated with virulence

Gene Disrupted	Gene name	Reference[a]
PSPTO_5516	*uvrD*	4
PSPTO_0917	*ndh*	5
PSPTO_4868	*retS*	6
PSPTO_3724	*lonI*	7
PSPTO_3576	*tvrR*	1
PSPTO_0965	*cbrB*	8
PSPTO_3349	*ftsK*	9
PSPTO_5483	*phoU*	10

a. Reference that describes how that gene is involved in virulence of *P. syringae* pv. tomato DC3000, similar plant-pathogenic bacteria, or a related *Pseudomonas* species.

Table S3: Primers and plasmids used in this study

Primer name	Sequence (5' – 3')	Description
oSWC01139	GTAACACTGGCAGAGCATTACGCTG	1st round of PCR 3' end of Tn
oSWC02330	CCTTTGCCATGTTTCAGAAACAACT	1st round of PCR 5' end of Tn
oSWC00141	GGCCACGCGTCGACTAGTACNNNNNNNNNNGAACG	Used during 1st round of PCR
oSWC00142	GGCCACGCGTCGACTAGTAC	Primer specific to oSWC00141 tail seq.
oSWC02331	GGATCAGATCACGCATCTTCCCGACA	2nd round of PCR 3' end nested primer for Tn
oSWC02332	GCAATGTAACATCAGAGATTTTGAG	2nd round of PCR 5' end nested primer for Tn
oSWC02209	ACCTACAACAAAGCTCTCATCAACC	Fwd sequencing primer
oSWC02210	GCAATGTAACATCAGAGATTTTGAG	Rev sequencing primer
**Plasmid**	**Description**	**Reference**
pΩ::*tvrR*	pΩ::*tvrR* Sm^R, Sp^R	1
pAC1	pΩ::*retS* Sm^R, Sp^R	2

1 Preiter, K. *et al.* Novel virulence gene of *Pseudomonas syringae* pv. tomato strain DC3000. *Journal of Bacteriology* **187**, 7805-7814, doi:10.1128/jb.187.22.7805-7814.2005 (2005).

2 Chambers, A. Characterization of the *Pseudomonas syringae* pathovar *tomato* DC3000 Rets hybrid two component sensor for induction of the type three secretion system and motility. (2010).

3 Consortium, T. U. UniProt: the universal protein knowledgebase. *Nucleic Acids Research* **45**, D158-D169, (2017).

4 Choi, J. Y. *et al.* Identification of virulence genes in a pathogenic strain of *Pseudomonas aeruginosa* by representational difference analysis. *Journal of Bacteriology* **184**, 952-961 (2002).

5 Granato, L. M. *et al.* The ATP-dependent RNA helicase HrpB plays an important role in motility and biofilm formation in *Xanthomonas citri* subsp. citri. *BMC Microbiology* **16**, 55 (2016).

6 Records, A. R. & Gross, D. C. Sensor kinases RetS and LadS regulate *Pseudomonas syringae* type VI secretion and virulence factors. *Journal of Bacteriology* **192**, 3584-3596, (2010).

7 Lan, L., Deng, X., Xiao, Y., Zhou, J. M. & Tang, X. Mutation of Lon protease differentially affects the expression of *Pseudomonas syringae* type III secretion system genes in rich and minimal media and reduces pathogenicity. *Molecular Plant-Microbe interactions : MPMI* **20**, 682-696, (2007).

8 Chakravarthy, S. *et al.* Virulence of *Pseudomonas syringae* pv. tomato DC3000 isinfluenced by the catabolite repression control protein Crc. *Molecular Plant-Microbe Interactions* **30**, 283-294, (2017).

9 Kinscherf, T. G., Hirano, S. S. & Willis, D. K. Transposon insertion in the *ftsK* gene impairs *in planta* growth and lesion-forming abilities in *Pseudomonas syringae* pv. syringae B728a. *Molecular Plant-Microbe Interactions* **13**, 1263-1265 (2000).

10 Chatnaparat, T., Prathuangwong, S. & Lindow, S. E. Global pattern of gene expression of *Xanthomonas axonopodis* pv. glycines within soybean leaves. *Molecular Plant-Microbe Interactions* **29**, 508-522, (2016).
